# Structural Basis for c-di-GMP-Mediated Inside-Out Signaling Controlling Periplasmic Proteolysis

**DOI:** 10.1371/journal.pbio.1000588

**Published:** 2011-02-01

**Authors:** Marcos V. A. S. Navarro, Peter D. Newell, Petya V. Krasteva, Debashree Chatterjee, Dean R. Madden, George A. O'Toole, Holger Sondermann

**Affiliations:** 1 Department of Molecular Medicine, College of Veterinary Medicine, Cornell University, Ithaca, New York, United States of America; 2 Department of Microbiology and Immunology, Dartmouth Medical School, Hanover, New Hampshire, United States of America; 3 Department of Biochemistry, Dartmouth Medical School, Hanover, New Hampshire, United States of America; Massachusetts General Hospital/Harvard Medical School, United States of America

## Abstract

The bacterial second messenger bis-(3′–5′) cyclic dimeric guanosine monophosphate (c-di-GMP) has emerged as a central regulator for biofilm formation. Increased cellular c-di-GMP levels lead to stable cell attachment, which in *Pseudomonas fluorescens* requires the transmembrane receptor LapD. LapD exhibits a conserved and widely used modular architecture containing a HAMP domain and degenerate diguanylate cyclase and phosphodiesterase domains. c-di-GMP binding to the LapD degenerate phosphodiesterase domain is communicated via the HAMP relay to the periplasmic domain, triggering sequestration of the protease LapG, thus preventing cleavage of the surface adhesin LapA. Here, we elucidate the molecular mechanism of autoinhibition and activation of LapD based on structure–function analyses and crystal structures of the entire periplasmic domain and the intracellular signaling unit in two different states. In the absence of c-di-GMP, the intracellular module assumes an inactive conformation. Binding of c-di-GMP to the phosphodiesterase domain disrupts the inactive state, permitting the formation of a trans-subunit dimer interface between adjacent phosphodiesterase domains via interactions conserved in c-di-GMP-degrading enzymes. Efficient mechanical coupling of the conformational changes across the membrane is realized through an extensively domain-swapped, unique periplasmic fold. Our structural and functional analyses identified a conserved system for the regulation of periplasmic proteases in a wide variety of bacteria, including many free-living and pathogenic species.

## Abstract 2

X-ray crystallographic structural analyses of the bacterial transmembrane receptor LapD identify conserved molecular mechanisms that control biofilm formation in response to changes in the intracellular levels of the second messenger c-di-GMP.

## Author Summary

Bacteria have the ability to form surface-attached communities, so-called biofilms, in both free-living environmental habitats and during pathogenic colonization in infectious diseases. Many of the cellular processes contributing to biofilm formation, for example, changes in motility, cell adhesion, and secretion, are regulated by the nucleotide-based second messenger c-di-GMP, which is unique to bacteria. In *Pseudomonas fluorescens*, there are high levels of c-di-GMP within the bacterial cell when there is plentiful nutrient availability inside the cell, and the c-di-GMP levels determine stable biofilm formation outside the cell. LapD, a transmembrane receptor for intracellular c-di-GMP, communicates changing c-di-GMP levels to the outside of the cell by controlling the stability of the large adhesin protein LapA, which keeps bacteria attached to a surface or to other cells. We conducted X-ray crystallographic analyses of the structure of the intracellular and periplasmic modules of LapD that, in combination with functional studies, including those shown in an accompanying study by Newell et al., reveal the molecular mechanisms regulating receptor function. When phosphate availability is severely restricted, intracellular c-di-GMP levels are low and LapD is in held in an “off” state by an autoinhibitory interaction, which permits the proteolytic processing of LapA, its release from the cell surface, and consequently biofilm dispersal. Conversely, when there are higher phosphate levels in the growth medium, c-di-GMP increases and binds to a cytoplasmic domain of LapD, disrupting the autoinhibitory state and triggering a conformational change that sequesters the periplasmic protease responsible for cleavage of LapA, ultimately yielding stable cell attachment. By revealing key motifs for the regulation of LapD, we have identified similar systems in many other bacterial strains that may control periplasmic protein processing events in a similar fashion.

## Introduction

Bacterial biofilms arise from planktonic microbial cells that attach to surfaces and form sessile multicellular communities, a process relevant to their survival in hostile habitats and for bacterial pathogenesis [1]. Recent work has identified biofilm formation as a multiphase process with strict temporal and spatial regulation, often accompanied by adaptational strategies such as phenotypic variation, development of antibiotic resistance, and virulence gene expression [2],[3]. On the cellular level, functional differentiation events including changes in motility, cell adhesion, and secretion are among the many processes driving bacterial biofilm formation. Such a plethora of physiological responses inevitably poses the question of how regulation is achieved, and a nucleotide unique to bacteria, bis-(3′–5′) cyclic dimeric guanosine monophosphate (c-di-GMP), has emerged as a key signaling molecule in this process [4],[5].

c-di-GMP is a monocyclic RNA dinucleotide that functions as an intracellular second messenger exerting control at the transcriptional, translational, and posttranslational levels [6]. It is generated from two guanosine triphosphate (GTP) molecules by GGDEF domain–containing diguanylate cyclases, and degraded by phosphodiesterases containing either EAL or HD-GYP protein domains [7]–[10]. The majority of cellular c-di-GMP appears to be bound to protein, eliciting localized, rather than more diffusive, signals [5]. To date, only a few c-di-GMP receptors have been identified, but they are strikingly diverse, including a class of riboswitches [11]. Protein domains involved in c-di-GMP signal recognition include PilZ domains [12],[13], a non-canonical receiver domain in VpsT of *Vibrio cholerae* [14], the AAA σ54 interaction domain–containing transcription factor FleQ of *P. aeruginosa* [15], and the cyclic nucleotide monophosphate–binding domain in Clp of *Xanthomonas campestris* [16]. In other cases, c-di-GMP turnover domains can also serve as sensors for the dinucleotide. For example, in GGDEF domain–containing proteins, an RxxD motif can serve as a c-di-GMP-binding inhibitory site either to regulate the activity of active enzymes (e.g., PleD of *Caulobacter crescentus* and WspR of *P. aeruginosa*) [17],[18] or to mediate protein–protein interactions in degenerate homologs (e.g., PelD of *P. aeruginosa* and CdgG of *V. cholerae*) [19],[20].

Bacterial proteins that mediate c-di-GMP turnover and signal transduction are often composed of multiple domains, allowing for a variety of regulatory inputs, signaling events, and/or physiological responses [21]. For example, a large number of these proteins contain both GGDEF and EAL domains in the same polypeptide chain. These proteins fall into three main categories based on their catalytic activity: tandem domain–containing proteins with both diguanylate cyclase and phosphodiesterase activity; proteins with only one active domain, in which the degenerate, inactive domain exhibits a regulatory function; and proteins in which both domains are degenerate and likely to work as c-di-GMP receptors [22],[23]. Despite the frequent occurrence of this signaling module in bacterial genomes, structural and mechanistic insight regarding their function and regulation is sparse.

The transmembrane protein LapD belongs to the last group. It contains degenerate GGDEF and EAL domains that lack catalytic activity, but it is capable of c-di-GMP binding via its divergent phosphodiesterase domain [24]. LapD is required for stable cell attachment and biofilm formation in *P. fluorescens* and *P. putida* [25]–[27]. It responds to changes in cellular c-di-GMP levels modulated by the availability of inorganic phosphate, an essential nutrient that is limiting in many ecosystems [24],[28]. Under phosphate starvation conditions, the expression of the phosphodiesterase RapA is upregulated, reducing cellular c-di-GMP levels and cell attachment. Increased phosphate availability yields an inactive Pho regulon, reduced RapA expression, and, as a consequence, a rise in cellular c-di-GMP concentration. As c-di-GMP levels change LapD switches between two states: the dinucleotide-unbound off state that retards stable biofilm formation by facilitating the secretion of the cell surface adhesin LapA, and the c-di-GMP-bound on state that supports cell adhesion by preventing the release of LapA from the outer membrane [24],[26]. Binding of c-di-GMP to the LapD EAL domain is relayed to the periplasmic output domain through an inside-out signaling mechanism that utilizes a juxtamembrane HAMP domain, a relay module often found in bacterial transmembrane receptors [24].

Accompanying work by Newell et al. [29] reveals the complete c-di-GMP signaling circuit by which LapD controls cell attachment in response to phosphate availability. For wild-type LapD, c-di-GMP binding appears to induce a conformational change, which activates the receptor. As a consequence, the affinity of the periplasmic domain for the cysteine protease LapG increases, limiting its access to LapA. Perturbations in the HAMP domain by deletion of some key elements yield a constitutively active receptor, independent of dinucleotide binding. However, it has remained unclear what prevents LapD from adopting an active conformation and how dinucleotide binding translates into an output signal.

Here, we present three crystal structures of LapD from *P. fluorescens* that provide models for the c-di-GMP-unbound cytoplasmic domain lacking only the HAMP domain, a c-di-GMP-bound EAL domain dimer, and the periplasmic domain. Together these structures span almost the entire receptor and elucidate molecular mechanisms that regulate LapD function. The crystal structure of the cytoplasmic module containing the GGDEF–EAL tandem domains reveals the presence of an autoinhibitory motif formed by a helical extension of the HAMP domain. In this inactive state, the GGDEF domain restricts dinucleotide access to the EAL domain module. The crystal structure of dimeric, c-di-GMP-bound EAL domains provides insight into the conformational changes resulting from dinucleotide binding. Based on the crystal structure of the periplasmic output domain of LapD, we identify functionally important residues and propose a model for the regulation of LapD activity in inside-out signal transduction. Finally, our structural studies highlight many conserved features that allow us to identify similar signaling systems in a variety of bacterial strains including common pathogens such as *V. cholerae* and *Legionella pneumophila*.

## Results/Discussion

### Inactive State of the Intracellular Module of LapD

**Figure 1:**
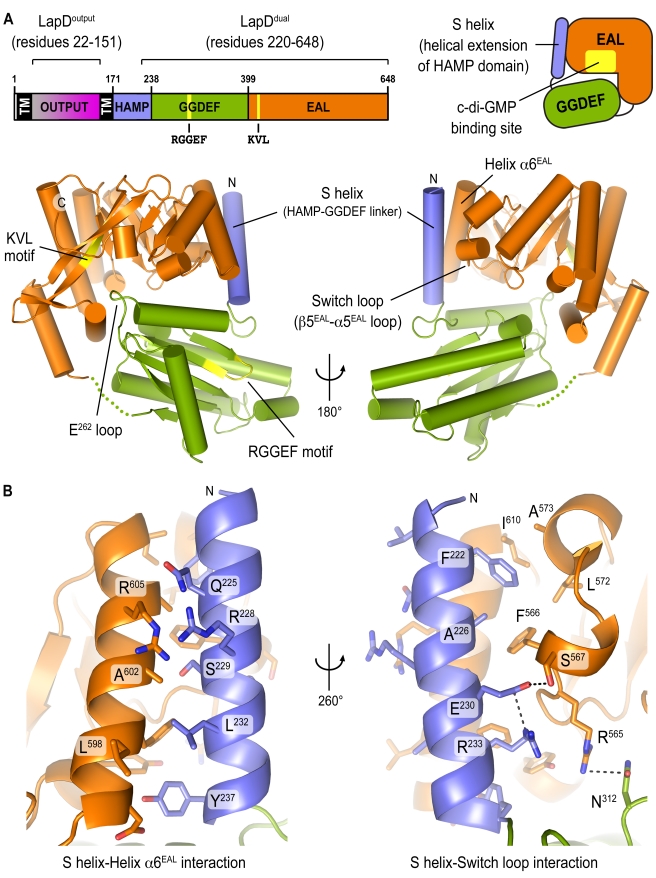
Autoinhibited structure of the cytoplasmic domain of LapD. (A) Crystal structure of apo-LapD^dual^. The domain organization of LapD from *P. fluorescens* Pf0-1 is shown. The degenerate sequence of the GGDEF and EAL signature motifs are indicated. The crystal structure of the LapD^dual^ (residues 220–648) is shown as ribbon presentation and colored according to the domain diagrams (upper panel). The S helix forms an extension of the second HAMP domain helix. The switch loop is sensitive to the nucleotide-binding state of the EAL domain and is involved in dimerization and catalysis in active phosphodiesterases. Two views, separated by a 180° rotation, are shown. (B) The S helix–EAL domain interface. A close-up view of the S helix–EAL domain interface is shown, with residues involved in direct, pairwise interactions shown as sticks. Two views, separated by a 260° rotation, are shown. Helix α6 and the switch loop form a surface buttressing the S helix.

In order to elucidate the molecular mechanism that regulates LapD function, we determined the crystal structure of the intracellular module of *P. fluorescens* LapD, comprising a HAMP–GGDEF domain linker segment and the degenerate GGDEF–EAL domain module (LapD^dual^; residues 220–648) (Figure 1). Based on secondary structure predictions, the linker forms a continuation of the second HAMP domain helix (Figure S1). We will refer to this motif as the signaling helix (S helix) in analogy to helical extensions found in association with other HAMP domains, where they are involved in transducing signals through the HAMP domain to the adjacent signaling modules [30]–[32].

The structure of LapD^dual^ (space group *P*3_2_, one molecule in the asymmetric unit) was solved by single-wavelength anomalous dispersion phasing using selenomethione-substituted protein crystals (Table S1). We also obtained a second crystal form involving different crystal packing contacts (space group *I*23, one molecule in the asymmetric unit), yet the overall structure of LapD^dual^ in the two crystals is identical (root mean square deviation [rmsd] of 0.9 Å over all atoms; Figure S2A and S2C). In both cases, the biologically significant unit was predicted to be a monomer, based on energetic and geometric estimations [33].

The overall fold of the GGDEF and EAL domains in LapD is very similar to those of other active or inactive diguanylate cyclases and phosphodiesterases, respectively (GGDEF domains: PleD, WspR, and FimX, average Cα–rmsd of 1.2 Å; EAL domains: YkuI, BlrP1, FimX, and TDB1265, average Cα–rmsd of 1.6 Å; Figures S3 and S4) [17],[18],[34]–[37]. The GGDEF signature motif in LapD consists of residues RGGEF, placing a glycine residue at the position of the active site residue that coordinates a divalent cation important for catalysis in active cyclases [38] (Figure S3). In addition, a non-conservative substitution introduces a charge change in another metal-coordinating residue in PleD (D^327^), which is an arginine residue in LapD (R^273^). Other significant changes that affect activity concern positive residues in PleD that interact with the phosphate moiety of GTP (K^442^ and K^327^). In LapD, these residues are glutamates (E^388^ and E^392^). In general, changes rendering LapD inactive for cyclase activity are comparable to those observed in FimX [36]. Similarly, the EAL domain of LapD contains non-conservative changes in residues important for catalysis (Figure S4). Most strikingly, the first residue of the signature EAL motif, which is involved in the coordination of a metal ion, is mutated to a lysine residue in LapD (KVL motif) [34],[35],[37],[39].

LapD^dual^ adopts a compact, bilobal conformation (Figure 1A). The GGDEF domain, comprising the N-terminal lobe, caps the dinucleotide-binding pocket of the EAL domain, which forms the C-terminal lobe of the tandem domain structure. The EAL domain buttresses the N-terminal S helix via predominantly hydrophobic interactions, burying 1,170 Å^2^ (Figure 1). The binding groove on the EAL domain, which accommodates the S helix, consists of the helix α6 and an adjacent loop. The latter has been identified as a conserved motif in catalytically active EAL domain–containing phosphodiesterases, in which it is involved in dimerization and catalysis [34],[40]. In LapD, the consensus sequence of the loop determined for active phosphodiesterases is not conserved [40]. This loop was referred to as loop 6 in SadR/RocR [40] and β5-α5 loop in the light-regulated phosphodiesterase BlrP1 [34]. We will refer to this motif as the switch loop of LapD, in analogy to the switch regions in G proteins.

In addition to the S helix–EAL domain interaction, the GGDEF domain contacts the dinucleotide-binding surface of the EAL domain at multiple points, forming a loosely packed interface that buries 1,620 Å^2^ of surface area (Figures 1A, S5A, and S5B). One such contact, the salt bridge between an arginine residue (R^450^) and a glutamate residue (E^262^), forms a particularly close interaction (Figure S5A). R^450^ is located just downstream of the signature EAL motif (KVL in LapD) at the center of the c-di-GMP-binding site. E^262^ is presented by a loop of the GGDEF domain. While E^262^ directly occupies the dinucleotide-binding site, the loop itself is located at its periphery, partially blocking access of c-di-GMP to the EAL domain (Figure S5B). Although the conformation of apo-LapD observed in the crystal structure is incompatible with c-di-GMP binding, the binding site is not completely occluded (Figure S5B), and there may be a sufficient proportion of accessible EAL domains in solution to respond to increasing c-di-GMP concentrations, competing with the inhibitory interactions. In addition, there may be cooperative effects within the dimeric, full-length receptor that are not apparent from the structures of the isolated domains.

The loop that connects the S helix to the GGDEF domain adopts a conformation that is identical to the linkage between active diguanylate cyclase domains and their regulatory domains (Figure S5C). The conformation is stabilized by a salt bridge between two strictly conserved residues that are located at the beginning of the connecting loop and just upstream of the signature GGDEF motif (^318^RGGEF^322^ in LapD), respectively: D^239^ in the loop and R^316^ in the GGDEF domain of LapD, D^174^ and R^249^ in WspR, and D^292^ and R^366^ in PleD [17],[18],[38],[41]. This interaction likely constrains the loop conformation, restricting the overall rotational freedom of the GGDEF domain relative to its associated regulatory module, the S helix in the case of LapD and the response receiver domain in the case of PleD and WspR.

In summary, the structural analysis of the cytoplasmic domain of LapD reveals that in the absence of c-di-GMP, the protein resides in a conformation incompatible with dinucleotide binding, with the GGDEF domain restricting access of c-di-GMP to the EAL domain. Dinucleotide binding would be accompanied by a major conformational change disrupting the conformation observed in the crystal structure.

### Crystal Structure of LapD^EAL^•c-di-GMP

**Figure 2:**
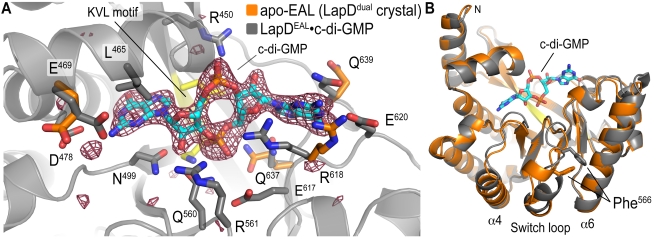
Comparison between the dinucleotide-free and c-di-GMP-bound EAL domain of LapD. (A) Crystal structure of LapD^EAL^•c-di-GMP. The c-di-GMP-bound structure of LapD^EAL^ (gray) was superimposed onto the dinucleotide-free structure of LapD^dual^ (orange residues). The S helix and GGDEF domain were omitted for clarity. A close-up view of the dinucleotide-binding pocket is shown, with residues involved in c-di-GMP binding presented as sticks. The (|*F*_o_| − |*F*_c_|) electron density map is shown as calculated from a model prior to inclusion of dinucleotide and is contoured at 3.5σ. (B) Conformational change of the switch loop. c-di-GMP binding and absence of the S helix allow the switch loop to adopt an alternative conformation (orange: apo-LapD^dual^; gray: LapD^EAL^•c-di-GMP). As a consequence, the side chain of F^566^, a residue involved in both S helix interaction in LapD^dual^ and dimerization of LapD^EAL^, changes position.

The crystal structure of LapD^EAL^ bound to c-di-GMP (residues 399–648; LapD^EAL^•c-di-GMP; Figure 2) was solved by molecular replacement using the EAL domain from apo-LapD^dual^ as the search model (Table S1). We obtained crystals in two independent conditions, yielding two different crystal forms (space group *C*222_1_, two molecules per asymmetric unit; and space group *P*6_5_22, one molecule per asymmetric unit; Figure S2B and S2C). While the majority of the crystal packing contacts were different, both crystal forms maintained a common dimer of EAL domains, and the resulting structures superimposed almost perfectly (rmsd of 0.6 Å over all atoms). Structures of the apo-EAL domain or c-di-GMP-bound LapD^dual^ could not be obtained to date, and the structural comparison will be made between the isolated EAL domain bound to c-di-GMP and apo-LapD^dual^.

c-di-GMP binding did not alter the overall conformation of the EAL domain observed in the apo-LapD^dual^ structure (rmsd of 0.6 Å over all atoms) (Figure 2), consistent with the lack of major conformational changes upon dinucleotide binding to the EAL domains of YkuI, TDB1265, and FimX [35]–[37]. Minor changes in the dinucleotide-binding pocket are confined to four c-di-GMP-coordinating residues that adopt an alternate side chain rotamer conformation (Figure 2A).

**Figure 3:**
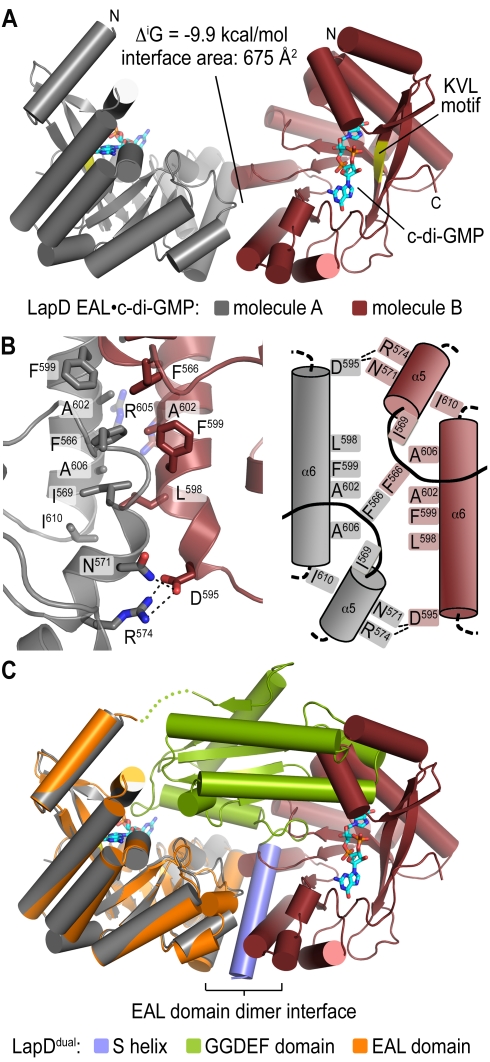
Dimerization of c-di-GMP-bound LapD^EAL^. (A) EAL domain dimerization. In both crystal forms obtained for LapD^EAL^•c-di-GMP we observe symmetric dimerization between protomers involving helix α6 and the switch loop. Dimerization buries 1,350 Å^2^ of surface area (interface area times two), and was predicted to be energetically favorable [33]. (B) Dimer interface. A close-up view (left panel) and cartoon diagram (right panel) of the dimer interface is shown. (C) Comparison of apo-LapD^dual^ and LapD^EAL^•c-di-GMP. The EAL domain from the crystal structure of dinucleotide-free LapD^dual^ was superimposed on one c-di-GMP-bound EAL domain from dimeric LapD^EAL^. LapD^dual^ is colored as shown in Figure 1.

The most notable conformational change in LapD^EAL^ upon c-di-GMP binding occurs in the switch loop (Figure 2B). Dinucleotide binding and the absence of the S helix in the isolated EAL domain allow the loop to restructure, resulting in the switching of the conserved phenylalanine residue F^566^ (Figure 2B). In apo-LapD^dual^, the side chain of F^566^ faces inward and is located at the center of the S helix–binding interface (Figure 1B). In contrast, the switch loop adopts a conformation in the c-di-GMP-bound structure positioning F^566^ so that it can participate in homodimerization (Figures 2B and 3). Whether this change is due to the flexibility of the loop, adjusting its conformation to accommodate the S helix–bound and dimeric states, or depends on dinucleotide binding awaits further structural analysis.

The symmetric LapD^EAL^ domain dimer is reminiscent of the oligomeric state in active EAL domain–containing phosphodiesterases, such as in *P. aeruginosa* SadR/RocR, *Bacillus subtilis* YkuI, *Thiobacillus denitrificans* TDB1265, and the BLUF domain–regulated photoreceptor BlrP1 from *Klebsiella pneumoniae*, where dimerization is involved in positioning an aspartate residue that in the active protein coordinates a cation for efficient catalysis (Figure S4) [34],[35],[37],[40]. Most importantly, dimerization of the c-di-GMP-bound EAL domains is incompatible with the conformation observed in the crystals of apo-LapD^dual^ (Figure 3C). The surface occupied by the S helix overlaps significantly with the homodimerization interface, which indicates that dinucleotide-induced conformational changes will include the displacement of the GGDEF domain and the S helix. More generally, the preservation of EAL domain dimerization in LapD and the conformational change of the switch loop upon c-di-GMP binding suggest their importance for signaling and regulation in GGDEF–EAL domain–containing proteins.

### Analysis of the Regulatory Mechanisms of LapD in Solution

Based on the crystallographic data, a simple model would suggest that LapD is subject to an autoinhibition mechanism. In contrast to other c-di-GMP receptors with known structures, in which the dinucleotide-binding site is freely accessible in the apo state (Figure S6), intramolecular interactions restrict dinucleotide access to the EAL domain in LapD. c-di-GMP binding would disrupt these interactions, resulting in a change in conformation of the receptor. Alternatively, mutations in the regulatory features predicted to destabilize the interaction should relieve the autoinhibition and alter the shape and activity of the receptor.

**Figure 4:**
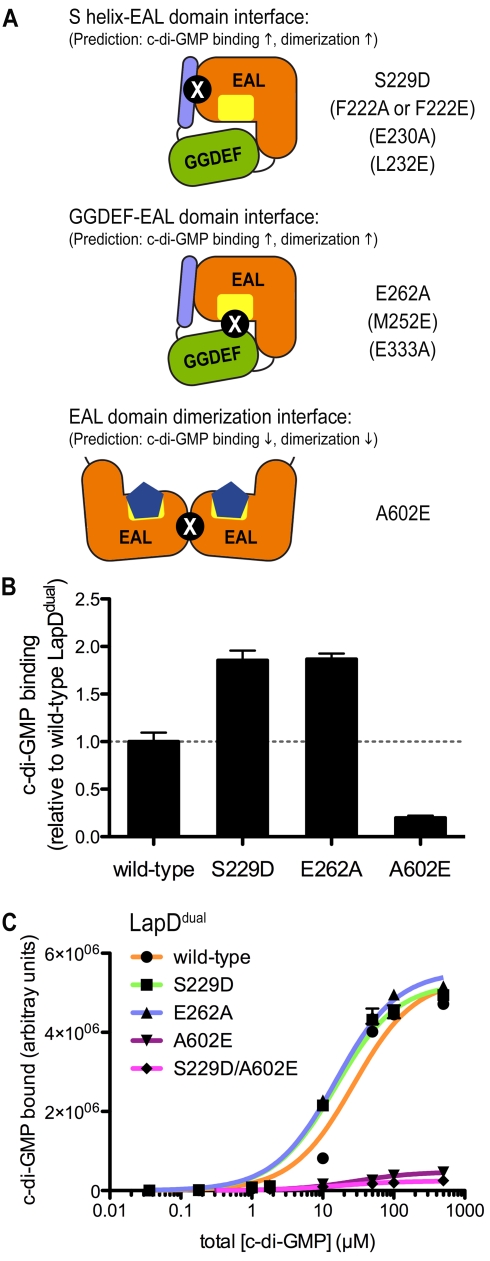
c-di-GMP binding of LapD^dual^ in solution. (A) Mutant categories. Structure-guided, site-directed mutants in LapD are illustrated. Mutations in brackets were used in experiments shown in Figures 8 and 9. Structure-based predictions regarding the c-di-GMP binding and c-di-GMP-dependent dimerization propensities are indicated. (B) Dinucleotide binding to wild-type and mutant LapD^dual^. Purified LapD^dual^ (wild-type, S^229^D, E^262^A, or A^602^E) was incubated in the presence of c-di-GMP. Excess dinucleotide was removed by gel filtration, and protein-bound c-di-GMP levels were assessed by reverse-phase HPLC after heat denaturation. Data are expressed relative to the amount bound to wild-type LapD^dual^. Data are means ± standard deviation (SD) of three independent experiments. (C) Filter binding assay. The amount of radiolabeled c-di-GMP bound by wild-type LapD^dual^ and mutant variants is plotted against the concentration of c-di-GMP. Data are means ± SD of three independent experiments. Table 1 summarizes the apparent *K*_d_ values obtained by applying a one-site-specific binding model.

To test this model, structure-guided mutations were introduced into LapD to assess the functional relevance of the autoinhibitory conformation and EAL domain dimerization (Figure 4A). Site-directed mutations were introduced into the S helix that were predicted to weaken its interaction with the EAL domain without affecting EAL domain dimerization propensity (F^222^A, F^222^E, S^229^D, E^230^A, or L^232^E; Figure 1B). Another set of mutations targeted the GGDEF–EAL domain interface, focusing on changes in the GGDEF domain that would not interfere with EAL domain function (M^252^E, E^262^A, or E^333^A; Figure S5A). Finally, A^602^ was targeted for mutation. A^602^ was identified as a residue at the center of the EAL domain dimerization interface (Figure 3B). The structure of apo-LapD^dual^ showed A^602^ at the periphery of the S helix–EAL domain interaction, suggesting that perturbations at this site may maintain the autoinhibited state (Figure 1B).

**Table 1:** Apparent affinity of LapD^dual^ or LapD^EAL^ for c-di-GMP.

Protein	Mutation	Apparent *K*_d_ (µM)	*B*_max_ a,b
LapD^dual^	Wild-type	27.0±4.7	5.4×10^6^
	S229D	15.1±1.8	5.3×10^6^
	E262A	15.3±1.7	5.6×10^6^
	A602E	>1 mM	n.d.
	S229D/A602E	>1 mM	n.d.
LapD^EAL^	Wild-type	13.1±0.9	5.5×10^5^
	A602E	36.3±5.4	5.6×10^5^

a For LapD^dual^ variants containing the A^602^E mutation, maximum binding at the highest c-di-GMP concentrations was significantly lower compared to LapD^dual^ lacking this point mutation. Assuming similar binding capacity of the various proteins, the data of the A^602^E-containing constructs of LapD^dual^ could not be fitted accurately, and estimated *K*_d_ values are much larger than the highest c-di-GMP concentration used in the titrations.

b LapD^EAL^ shows an overall weaker dimerization propensity than LapD^dual^, which affects the stability of the nucleotide-bound state.

n.d., not determined.

Mutations were introduced into LapD^dual^, the EAL domain, and the full-length receptor. It is important to note that LapD is a dimeric receptor via its HAMP and output domains, and therefore EAL domain dimerization (and dinucleotide binding) represents a conformational change within the receptor, rather than a change in its oligomeric state. The comparative analyses described below reveal the basic properties of the cytoplasmic module of LapD, especially the correlation between c-di-GMP binding and dimerization (Figures 4–7). However, the specific interaction energies will likely be enhanced in the context of the full-length receptor compared to those of the isolated domains. Cell-based assays elucidate the functional relevance of these properties in intact LapD (Figures 8 and 9).

**Figure 5 pbio-1000588-g005:**
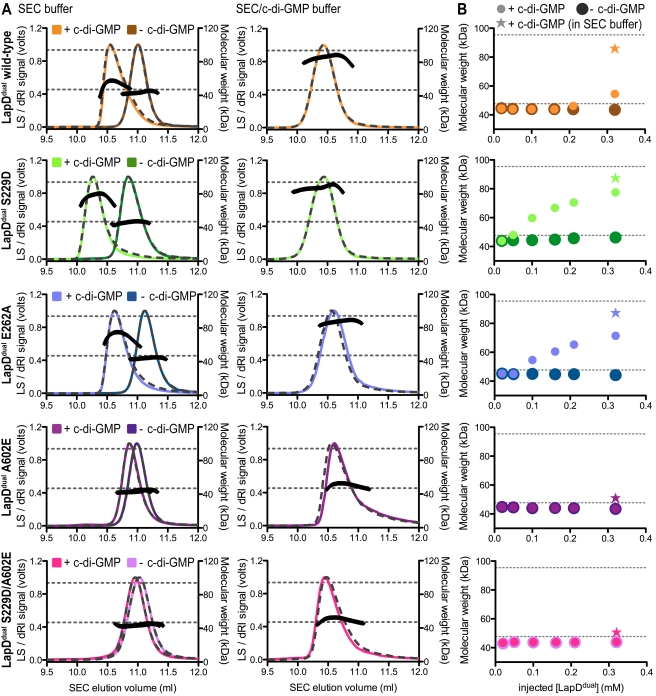
Quaternary state of LapD^dual^ in solution. (A) Oligomerization of LapD^dual^ in solution. SEC-coupled MALS analysis of wild-type and mutant LapD^dual^ in the presence and absence of c-di-GMP is shown. The signal from the 90° scattering detector is shown in color, and the signal from the refractive index detector is shown as a dashed line. Average molecular weights are plotted in black against the right *y*-axis as calculated every second across the protein elution peak. Theoretical molecular weights corresponding to those of a monomer and a dimer are indicated as horizontal dashed gray lines. Injected protein and dinucleotide concentrations were 250 µM and 500 µM, respectively. In the right panel, the mobile phase contained c-di-GMP (50 µM). Earlier elution times may indicate a more elongated conformation of certain mutants in solution (for example, of the mutant S^229^D compared to wild-type or the E^262^A variant in the absence of c-di-GMP), which is probably due to a displacement of the GGDEF domain from the EAL domain. (B) Concentration-dependent dimerization of LapD^dual^. SEC-MALS experiments were carried out with samples of increasing LapD^dual^ concentration. The samples of highest concentration correspond to data shown in (A). The data point shown as a star represents data obtained for samples run in a mobile phase that contained c-di-GMP.

**Figure 6 pbio-1000588-g006:**
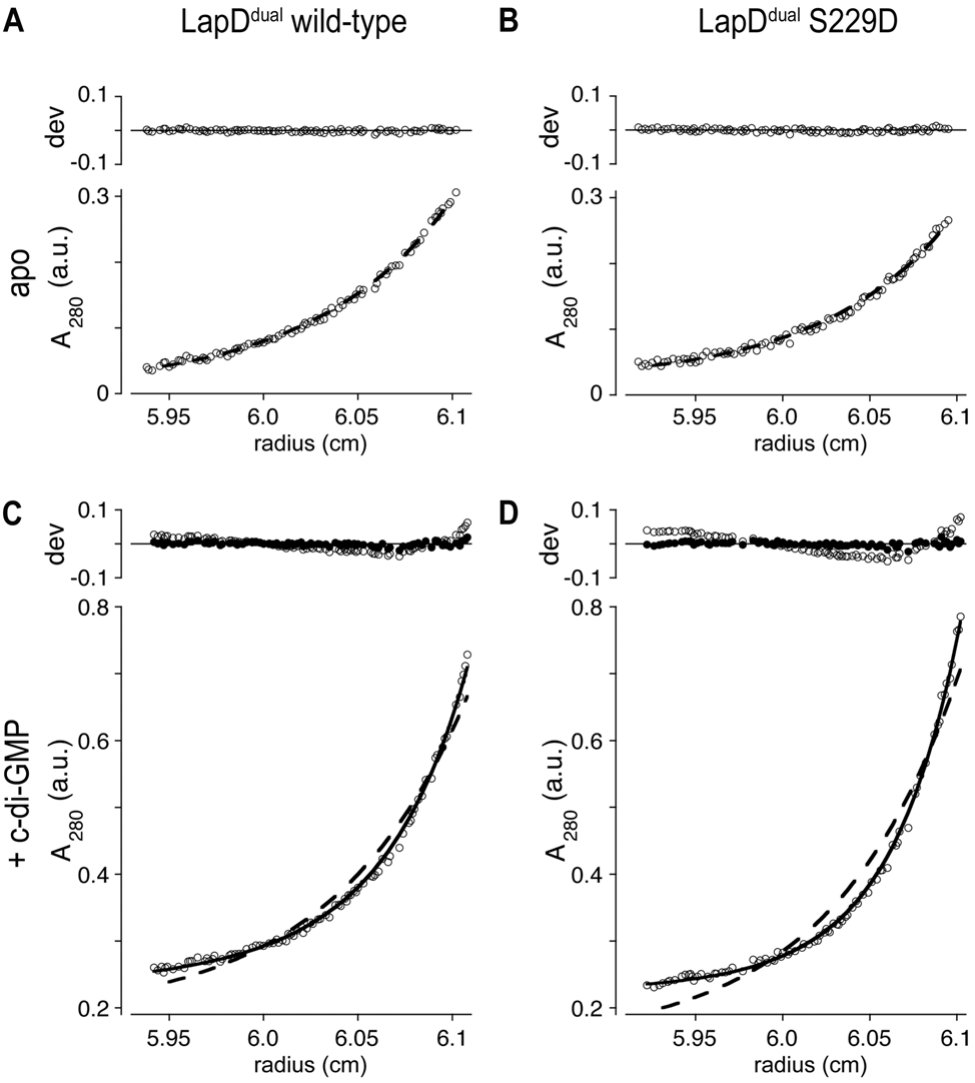
Sedimentation equilibrium analysis of LapD^dual^ dimerization. Experimental sedimentation equilibrium absorbance profiles (A_280_, open circles) are shown for wild-type (A and C) and S^229^D mutant (B and D) forms of LapD^dual^, in the absence (A and B) and in the presence (C and D) of 20 µM c-di-GMP. Data are shown for the lowest-concentration channel (∼3 µM protein) following equilibration at 20,000 rpm. The corresponding curves predicted by three-speed, three-channel global fits are shown for monomer-only (dashed line, [A–D]) and monomer:dimer equilibrium (solid line, [C and D]) models. The deviation between observed and calculated A_280_ values is shown above each profile for the monomer-only (open circles, [A–D]) and for the monomer:dimer equilibrium (closed circles, [C and D]) models. Systematic deviations between the monomer-only prediction and the experimental data in the presence of c-di-GMP are resolved by inclusion of a dimerization equilibrium for the c-di-GMP-bound form of LapD^dual^ (solid curves). a.u., absorbance units.

**Figure 7:**
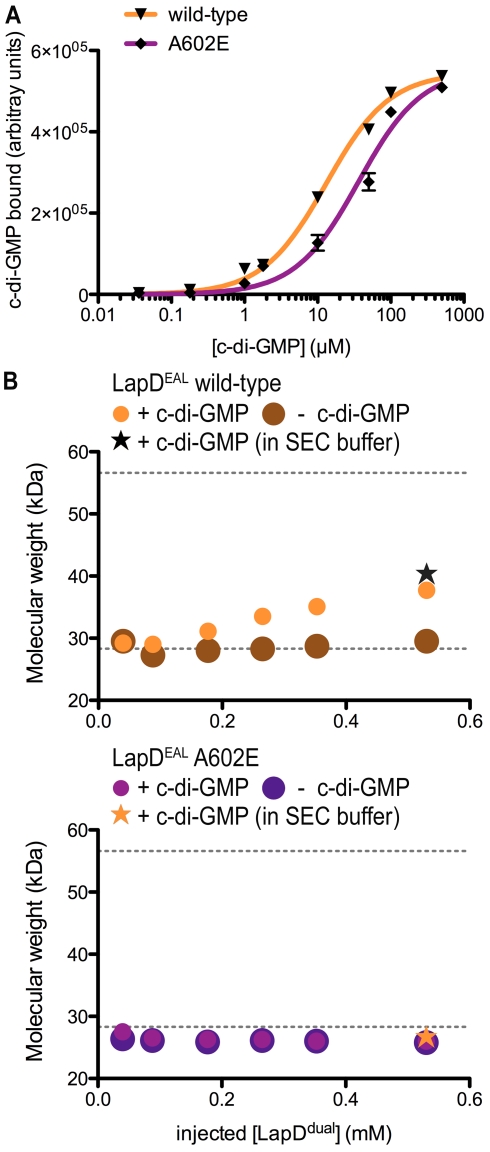
c-di-GMP binding and quaternary state of LapD^EAL^ in solution. (A) c-di-GMP binding. The amount of radiolabeled c-di-GMP bound by LapD^EAL^ (wild-type or A^602^E) is plotted against the concentration of c-di-GMP. Data are means ± SD of three independent experiments. Data were fitted to a one-site-specific binding model. (B) Oligomerization in solution. SEC-MALS analysis of wild-type and mutant LapD^EAL^ in the presence and absence of c-di-GMP at increasing protein concentration is shown. The protein molecular weight was determined based on the intensity of the scattered light at multiple angles. Theoretical molecular weights corresponding to those of a monomer and a dimer are indicated as horizontal dashed gray lines. Injected protein and nucleotide concentrations were 250 µM and 500 µM, respectively. Experiments at the highest protein concentration were carried out in the absence (circles) or presence of c-di-GMP (star) in the mobile phase.

**Figure 8:**
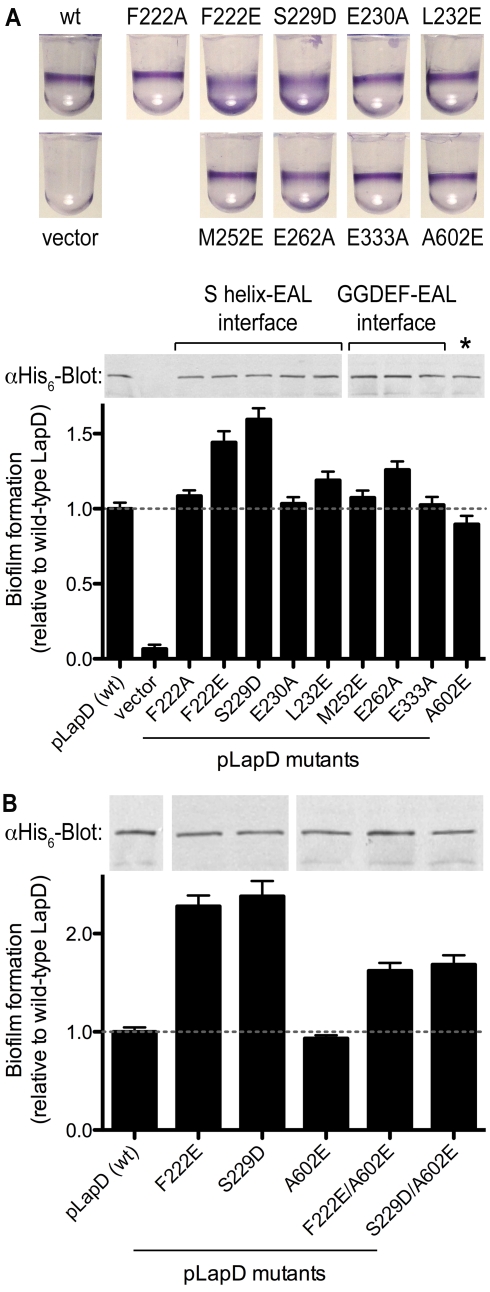
Phenotypic analyses of *lapD* mutants. (A) Biofilm phenotypes. Biofilm formation of Δ*lapD* cells expressing full-length, wild-type LapD, LapD point mutants, or the insert-less expression vector was assessed. Crystal violet-stained biofilms (top) and their quantification (bottom) are shown. Data are means ± SD of eight replicates. Protein levels were determined by Western blotting using a primary antibody that recognizes His_6_ epitope at the C-terminus of LapD. The asterisk marks a residue at the center of the EAL domain dimerization interface. (B) Biofilm phenotypes of double mutants. The analysis was carried out as described in (A). Data are means ± SD of eight replicates.

**Figure 9:**
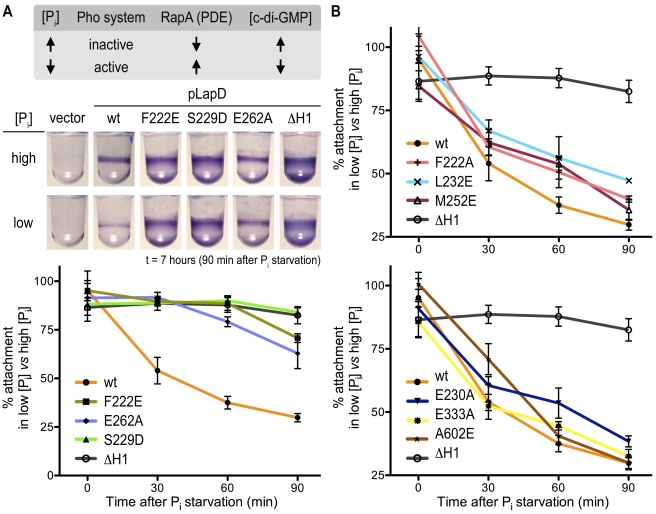
Phosphate-regulated c-di-GMP signaling via LapD. (A) Phosphate-regulated c-di-GMP signaling. Phosphate (P_i_) starvation leads to the expression of the active phosphodiesterase RapA and a reduction in cellular c-di-GMP concentration [24]. LapD mutants were tested for their response to limiting phosphate concentration. Biofilm formation was monitored over 90 min after physiological activation of the Pho system in low-phosphate medium, and compared to biofilm formation in phosphate-rich medium. The mutant ΔH1 contains an activating deletion in the HAMP domain and has been described previously [24]. Data are means ± SD of eight replicates. (B) Mutants showing intermediate responses. The analysis was carried out as described in (A). Data are means ± SD of eight replicates.

We employed two methods to assess c-di-GMP binding to LapD. A gel-filtration-based assay essentially measures the off rate of nucleotide from a preformed complex. The filter binding assay is a semi-quantitative assay that allows for higher throughput and the generation of titration curves yielding an apparent dissociation constant (*K*_d_) [24] (Table 1). Mutations in the regulatory motifs and dimer interface have a measurable effect on c-di-GMP binding to LapD. A single-point mutation in the S helix increased the overall dinucleotide binding and the apparent affinity of LapD^dual^ for c-di-GMP by almost 2-fold (S^229^D; Figure 4B and 4C). Removal of the glutamate side chain in residue 262 that occludes the dinucleotide binding site in the LapD^dual^ structure (E^262^A) has a similar effect. In contrast, replacing A^602^ with a glutamate residue reduced c-di-GMP binding to LapD^dual^ both in the gel-filtration-based binding experiment and in a filter binding assay, suggesting an interdependence of dinucleotide binding and EAL domain dimerization.

We next analyzed the oligomerization state of LapD^dual^ protein variants in solution, using static multi-angle light scattering (MALS) (Figure 5). This method provides the population-averaged absolute molecular weight and hence quaternary state of proteins eluting from a gel filtration column. The technique measures the intensity of scattered laser light from a particle at multiple angles, which is proportional to the product of the molecular weight and the concentration of the particle, permitting rapid and facile comparison of oligomeric equilibria across a series of mutants [42].

The wild-type LapD^dual^ protein elutes in a single peak from the size exclusion column with a molecular weight of 43.5 kDa, indicating a monomeric state in solution (Figure 5A, left column). Incubation of the protein with c-di-GMP shifted the peak elution volume and increased the molecular weight slightly to 54.5 kDa. While being monomeric in the absence of dinucleotide, both the S helix–EAL and the GGDEF–EAL interface mutants (S^229^D and E^262^A, respectively) showed more distinct shifts in molecular weight towards dimeric species upon c-di-GMP binding (77.5 kDa and 71.4 kDa, respectively; Figure 5A, left column). As predicted on the basis of the structural analysis, LapD^dual^ variants containing a glutamate substitution in place of A^602^ (A^602^E and S^229^D/A^602^E) are monomeric in solution, independent of the presence of dinucleotide and unaffected by the additional mutation S^229^D.

In general, the intermediate molecular weights and non-Gaussian peak shapes observed for wild-type LapD^dual^ and the mutants S^229^D and E^262^A, predicted to be less inhibited, incubated with c-di-GMP prior to gel filtration, may indicate a fast exchange between monomeric and dimeric species relative to the data acquisition time and/or instability of the complex. To further investigate this phenomenon, we conducted concentration-dependent experiments by subjecting LapD^dual^ to light scattering measurements at concentrations between 20 and 320 µM with or without incubation in c-di-GMP. All samples eluted as single peaks from the gel filtration column and showed no signs of unspecific protein aggregation. Protein concentration determination across the peak volume indicated that samples were diluted consistently ∼15-fold during the chromatography. All LapD^dual^ variants were monomeric in the absence of c-di-GMP across the entire concentration range (Figure 5B). LapD^dual^ proteins with a mutation at the dimerization interface (A^602^E or S^229^D/A^602^E) were insensitive to c-di-GMP addition and remained monomeric. Wild-type LapD^dual^ showed signs of oligomerization only at the highest concentrations tested. In contrast, the molecular weight of LapD^dual^ variants with single-point mutations S^229^D or E^262^A, predicted to disrupt autoinhibitory features, increased in a concentration-dependent manner in the presence of c-di-GMP, indicative of dimerization of the isolated cytoplasmic domain in solution.

Considering the modest dinucleotide-binding affinities (Figure 4B and 4C), dissociation of c-di-GMP from LapD during gel filtration may also contribute to a destabilization of the dimeric state. To investigate this possibility, we repeated the experiments at the highest protein concentration with c-di-GMP present in the mobile phase (Figure 5). While proteins containing the A^602^E mutation (A^602^E or S^229^D/A^602^E) remained monomeric, wild-type LapD^dual^ and the mutants S^229^D and E^262^A exhibited more pronounced dimerization in the same assay, with molecular weights close to the theoretical values for dimers calculated based their sequence. The observation that wild-type LapD^dual^ displayed only a moderate, c-di-GMP-induced dimer formation when c-di-GMP was omitted from the mobile phase, but robust dimerization when the dinucleotide was present throughout the experiment, distinct from the behavior of the mutants S^229^D or E^262^A, indicates that the c-di-GMP-induced conformational changes and dimerization are reversible and underscores the interdependence of dinucleotide binding and EAL domain dimerization (Figure 5).

In order to investigate the propensity for dimer interface formation under equilibrium conditions, we performed analytical ultracentrifugation experiments on wild-type LapD^dual^ and on the S^229^D mutant. As expected based on the light scattering analysis, the concentration profiles of the c-di-GMP-free proteins could be well fit by a monomeric model, assuming a fixed molecular weight equivalent to the calculated value (Figure 6A and 6B). When allowed to refine against a monomer:dimer equilibrium, the *K*_d_ for dimerization (*K*_d_^dimer^) refined to values of 400 µM (95% confidence interval: 0 to 6,300 µM) and 420 µM (95% confidence interval: 600 to 4,700 µM), respectively. Thus, each construct exhibits only a minimal propensity for dimerization, and apo-LapD^dual^ is statistically indistinguishable from a pure monomer population. However, and again consistent with the light scattering data, in the presence of c-di-GMP, the concentration profiles of both proteins were poorly modeled unless the bound state was allowed to form dimers (Figure 6C and 6D). In this case, the refined *K*_d_^dimer^ values were 670 nM (95% confidence interval: 370 to 1,000 nM) and 180 nM (95% confidence interval: 80 to 270 nM), respectively. It is thus clear that in the presence of c-di-GMP, the propensity of the intracellular domain to form a dimer interface is several orders of magnitude stronger than that of the apo states of both proteins. Based on the nonoverlapping confidence intervals, it also appears that there may be a slight, but statistically significant, enhancement in the dimerization propensity of the S^229^D mutant, paralleling its increased affinity for c-di-GMP and the results from the light scattering experiments.

By and large, comparable results were obtained for the isolated EAL domain (Figure 7; Table 1). The wild-type domain bound c-di-GMP with an apparent *K*_d_ of 13.1 ± 0.9 µM, whereas the A^602^E mutant showed a decreased affinity, with an apparent *K*_d_ of 36.3 ± 5.4 µM (Figure 7A). Similar to LapD^dual^, the isolated EAL domain showed concentration-dependent oligomerization in light scattering experiments only upon incubation with c-di-GMP (Figure 7B). The presence of c-di-GMP in the mobile phase stabilized the dimeric species further, although to a lesser extent then observed with LapD^dual^. In contrast, the EAL domain containing the A^602^E mutation remained monomeric even in the presence of c-di-GMP. In general, LapD^dual^ shows a higher propensity for dimer formation than LapD^EAL^ (Figures 5 and 7), and this behavior correlates with the stability of the nucleotide-bound complex (see above). The mutation A^602^E severely affects dimer formation of LapD^dual^, and hence nucleotide binding. Additionally, the contribution of the GGDEF domain to dimerization in LapD^dual^ would also be consistent with a larger apparent impact of the A^602^E mutation on dimerization. The effect of the A^602^E mutation is less pronounced for LapD^EAL^ since this construct forms weaker dimers overall. Together, these data suggest a similar mode of dimerization of LapD^EAL^ and LapD^dual^. However, in comparing the light scattering results in the presence and absence of c-di-GMP in the mobile phase, the greater discrepancy in residual dimerization observed for the LapD^dual^ construct suggests that in the tandem domain the autoinhibited structure reassembles as nucleotide is withdrawn.

In summary, LapD appears to be autoinhibited for efficient dinucleotide binding by structural features involving the S helix and occupancy of the c-di-GMP-binding site by the GGDEF domain. Based on the observation that the A^602^E mutation, located in the EAL domain homodimer interface and outside of the c-di-GMP-binding site, renders the protein monomeric and reduces dinucleotide binding, we propose that dimerization and c-di-GMP binding are interdependent events in LapD^dual^ and LapD^EAL^. An additional conformational change in the cytoplasmic domain of LapD, accompanied by the release of the inhibitory S helix and/or nucleotide binding, is likely to occur as well.

### Effect of Structure-Based Mutations in LapD on Biofilm Formation

Stable biofilms of *P. fluorescens* require LapD expression and the presence of c-di-GMP [24]. To examine the contribution of inter-domain interactions to LapD's function in vivo, full-length LapD variants were assessed for their ability to promote biofilm formation in a Δ*lapD* mutant strain (Figure 8). We observed a range of phenotypes, from a slight reduction in biofilm formation relative to the wild-type, to strong hyper-adherent phenotypes comparable to that observed when LapD is constitutively activated by mutations in the HAMP domain [24] (Figures 8 and 9). The mutation that we predict to disrupt the S helix–EAL interface in the autoinhibited conformation, S^229^D, caused an “activated” phenotype, consistent with its increased dinucleotide binding and dimerization propensity in vitro (Figures 4–6). Similar results were obtained with the mutant F^222^E, whereas a less disruptive alanine substitution was tolerated at this position.

In the apo-LapD^dual^ structure, the E^262^ residue is positioned such that it would occlude binding of c-di-GMP to the EAL domain (Figure S5B). Consistent with this and its increased binding of c-di-GMP (Figure 4), the E^262^A mutation results in an increase in biofilm formation relative to the wild-type allele (Figure 8A). Yet, the E^262^A mutant phenotype is not as extreme as that exhibited in the case of the S^229^D mutation, despite comparable increases in c-di-GMP binding and dimerization by these proteins in vitro (Figures 4 and 5). This suggests that the E^262^A mutant is still subject to autoinhibition in vivo, albeit with higher sensitivity for c-di-GMP than the wild-type protein. Structurally, this may be explained by removal of the side chain that directly occupies the c-di-GMP-binding site without disturbing the S helix–EAL domain interaction. Other mutations showed intermediate (L^232^E and M^252^E) or no significant changes (F^222^A, E^230^A, and E^333^A) in phenotype, roughly corresponding to their surface exposure in the autoinhibited state structure (Figures 1B, 8A, and S5A).

The A^602^E mutation, which disrupts the dimerization interface of the EAL domain and reduces steady state c-di-GMP binding in vitro (Figures 4, 5, and 7), led to a small but significant decrease in biofilm formation relative to the wild-type allele (Figure 8). The observation that the A^602^E mutant showed a minor loss of function in vivo, distinct from the more pronounced loss of function observed with mutants in the dinucleotide binding pocket [24], argues that dimerization increases the stability of the dinucleotide-bound state rather than being required for c-di-GMP binding per se. While this modest reduction in function in vivo seemed incongruous with the severe defect in dimerization and binding exhibited by the dual-domain and EAL domain construct in vitro, we further tested its significance by introducing the A^602^E mutation into activated alleles of LapD, S^229^D, and F^222^E. The reduction in biofilm formation in the double mutants was significant, corroborating that EAL domain dimerization plays a role in LapD function in vivo (Figure 8B).

The single mutants were also tested for their response to phosphate starvation, a physiological input for LapD-mediated signaling that leads to a reduction of cellular c-di-GMP concentration [24],[28]. At low c-di-GMP concentration, wild-type LapD activity is downregulated, which results in the release of the adhesin LapA from the cell surface and thus a reduction in biofilm formation (Figure 9A, top) [24]. Mutations in the S helix–EAL domain interface (F^222^E and S^229^D) failed to respond to phosphate starvation efficiently, showing little to no reduction in biofilm formation (Figure 9A). The effect was comparable to a deletion mutant described previously, in which a helical segment of the HAMP domain was removed, yielding a constitutively active, deregulated receptor (Figure 9A) [24]. In contrast, mutation of the residue in the GGDEF domain that occupies the c-di-GMP-binding site (E^262^A) showed an intermediate response to phosphate starvation, suggesting that mutant receptor function is still controlled by c-di-GMP, albeit not as effectively as in wild-type LapD (Figure 9A). Similar to the trends observed in the static biofilm assay (Figure 8A), other mutations in LapD showed more subtle effects in the phosphate starvation experiments (Figure 9B).

Collectively, these results suggest that the S helix–EAL domain interface stabilizes the off state. The interaction is the dominant autoinhibitory feature responsible for positioning the GGDEF domain to occlude the c-di-GMP-binding pocket and therefore ensure appropriate control of LapD activation in vivo. In addition, EAL domain dimerization via a conserved mode of interaction is likely to contribute to the efficiency of the signaling system by stabilizing the activated conformation, although it appears to be a secondary component of the activation mechanism.

### Crystal Structure of LapD's Output Domain: A Conserved, Domain-Swapped Periplasmic Domain

**Figure 10:**
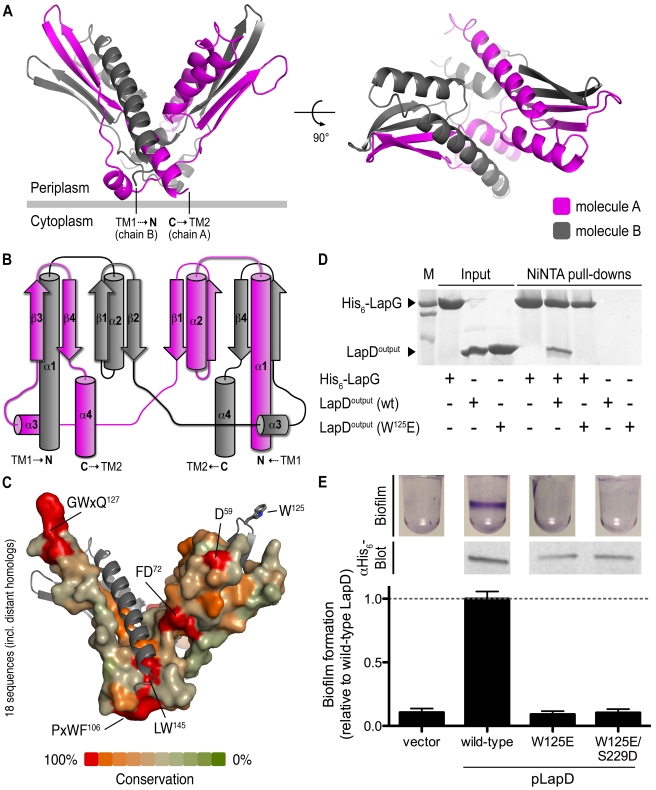
Structure–function analysis of the periplasmic output domain of LapD. (A) Crystal structure of LapD^output^. The crystal structure of the periplasmic output domain of LapD (residues 22–151) is shown as a ribbon presentation, with the two protomer chains colored in pink and gray, respectively. The relative position of the inner cell membrane (gray bar) and connection to the flanking transmembrane (TM) helices are indicated. Two orthogonal views are shown. (B) Topology diagram. The diagram illustrates the domain-swapped structure of the dimeric output domain. (C) Surface conservation. Based on an alignment of 18 sequences of LapD homologs, the sequence conservation was mapped onto the accessible surface of the output domain. One protomer is shown as a surface presentation, the other is shown as a ribbon presentation. Conserved motifs and individual residues are highlighted. (D) LapD^output^–LapG complex formation. Purified His_6_-tagged LapG (His_6_-LapG) was bound to NiNTA, and incubated in the absence or presence of untagged, wild-type LapD^output^, or a LapD^output^ mutant in which W^125^ has been replaced with a glutamate. The Coomassie-stained gel shows eluates of NiNTA-bound proteins. (E) Biofilm phenotypes and LapD stability. Biofilm formation of Δ*lapD* cells expressing full-length, wild-type LapD, LapD point mutants, or the insert-less expression vector was assessed. Protein levels are shown by Western blotting for the His_6_ epitope at the C-terminus of LapD.

In order to shed light on how changes in the cytosolic domain are sensed in the periplasm, we determined the structure of the entire output domain (residues 22–151; Figure 1A). Crystals grown with selenomethionine-derivatized protein diffracted X-rays to a maximum resolution of 1.8 Å (Table S1). The structure was solved by single-wavelength anomalous dispersion phasing. The final model consists of two molecules per asymmetric unit spanning residues 23–150 (Figures 10A and S7A).

The periplasmic output domain of LapD forms an extensively interwoven, domain-swapped dimer sharing 3,429 Å^2^ interfacial surface area between the protomers (1/3 of LapD's output domain molecular surface) (Figures 10 and S7B). The dimer adopts an overall V-shaped conformation. Each arm of the fold consists of two α-helices and two β-strands contributed by one of the two protomers, complemented by two β-strands flanked by helical segments from the other. The N- and C-terminal helices of LapD's output domain presumably connect directly to the transmembrane helices and the HAMP domains. The two half sites are linked via a long connecting segment that crosses over at the center of the dimer. The two protomers superimpose well except for a subtle rigid body rotation around the linker (Figure S7A).

A DALI (distance-matrix alignment) search comparing LapD's output domain to proteins in the RSCB Protein Data Bank (PDB) revealed structural similarity of its domain-swapped arms to the periplasmic domain of the sensor histidine kinase CitA (*Z*-score  =  5.4, rmsd of 2.5 Å) [43]–[45]. The periplasmic modules of CitA and related proteins show some homology to PAS domains and have been classified as PDC (PhoQ-DcuS-CitA) protein domains [46],[47]. Such domains occur in many other bacterial transmembrane proteins, but unlike LapD's output domain, they are found to form a variety of regular, non-swapped dimers [44],[47],[48].

A sequence alignment of 18 sequences was constructed, including LapD homologs from other *Pseudomonas* strains and extending to more distantly related sequences from other bacterial genera (Figure S1; Table 2). Mapping sequence conservation onto the accessible molecular surface revealed a few potentially important motifs (Figures 10C and S8. The PxWF and LW segments (residues 103–106 and 144–145 of LapD, respectively) form a continuous surface at the bottom of the dimer. While the LW segment is part of the surface that accommodates the long N-terminal helix of the adjacent protomer, the PxWF is likely to interact with the inner membrane. The other striking feature is a strictly conserved loop connecting the strands β3 and β4 formed by the conserved GWxQ motif (residues 124–127 of LapD). W^125^ forms the most distal point of the periplasmic domain located at the center of the loop, and its side chain is in an outward-facing rotamer conformation (Figure 10C).

**Table 2:** Conservation of the LapD/LapG signaling system.

Organism	LapG-Like	E-Value	LapD-Like	E-value	ABC Transporter	Putative Substrate	Description	Putative Cleavage Site
*Pseudomonas fluorescens*	PFL_0130	6E-146	PFL_0131	0	X	PFL_0133	LapA homolog	QQAIAAGVDPTTALESTAAGPSAAGTGGAA
*Pseudomonas putida*	PP_0164	7E-95	PP_0165	0	X	PP_0168	LapA homolog	QQAIAAGVDPTTELEATAAGPSSAGGGAL
*Pseudomonas entomophilia*	PSEEN0138	4E-93	PSEEN0139	0	X	PSEEN0141	LapA-like adhesin	QQAIAAGADPTTELEATAAGPAAAGGGSV
*Erwinia carotovora*	ECA3263	3E-66	ECA3264	0	X	ECA3266	LapA-like adhesin	QDAIAQGADPTQVLEATAAGNDNTGEAG
*Pseudomonas aeruginosa*	PA1434	9E-66	PA1433	0	?	?	?	?
*Desulfotalea psychrophila*	DP0518	1E-47	DP0517	1E-83	X	DP0516	Large RTX protein	QALAEGKSIDDVLEKTAAGTEGSGGSYDF
*Bordetella parapertussis*	BPP0973	3E-45	BPP0972	3E-56	X	BPP0974	Hemagglutinin-like protein	LAALQDGRDPFDELDPTAAVIGGSGDSAGS
*Chromobacterium violaceum*	CV_0309	3E-45	CV_0310	7E-56	X	CV_0311	LapA-like adhesin	QQILAALDNPSANPGNPFDNLDPAAAGLND
*Vibrio cholerae El Tor*	VC_A1081	7E-43	VC_A1082/83	2E-36/3E-14	X	VCA0849	Hemagglutinin-like protein	QQAILDGVDPTTALEAAAAGAGAGG
*Bordetella bronchiseptica*	BB1185	3E-45	BB1184	2E-56	X	BB1186	Hemagglutinin-like protein	QDGRDPFDELDPTAAVIGGSGDSAGS
*Azoarcus* sp. *EbN*	ebA1785	4E-45	ebA1787	5E-83	X	ebA1795	Hypothetical protein	NQIIEAINEGANLDDVLEAPAAGLAGGGGG
*Vibrio cholerae 1587*	A55_A0980	8E-43	A55_A0981	7E-54	?	A55_A0690	TP1SS repeat 143	QQAILDGVDPTTALEAAAAGAGAGG
*Rhodoferax ferrireducens*	Rfer_3763	1E-42	Rfer_3764	3E-90	X	Rfer_3766	Hemolysin-type RTX protein	IQALERGTDLSTELEATAAGLGVGGG
*Shewanella denitrificans*	Sden_0379	1E-42	Sden_0378	2E-52	X	Sden_0384	Large RTX protein	QDLIASGEDPTEDLPETAAGTPTGNQGNS
*Vibrio fischeri*	VF_A1167	3E-42	VF_A1166	3E-56	X	VF_A1162	FrpC-like RTX protein	LNAIIDGEDPSLITEAPAAGEDSGS
*Vibrio vulnificus CMCP6*	VV2_1126	3E-41	VV2_1127	2E-54	X	VV1_2715	RTX protein	QQIFAALEEGQDPTQLGDDFATAAGETGG
*Thiomicrospira crunogena*	Tcr_0209	5E-41	Tcr_0208	2E-78	X	Tcr_0105	VCBS protein	EEIRKAIEAVQEGNFDALEETAAGQQNDL
*Photobacterium profundum*	PBPRB0581	5E-41	PBPRB0580	3E-56	X	PBPRB0585	Hypothetical protein	QNAILSGDDPTETLDAAAAGNEAQGSS
*Polaromonas* sp.	Bpro_0309	2E-40	Bpro_0308	3E-92	X	Bpro_0306	Large RTX protein	QALERGTDLNQSLEATAAGLVPGGG
*Legionella pneumophila lens*	lpl0859	3E-40	lpl0860	4E-81	?	lpl0681	RtxA/TP1SS repeat 143	QEAIAKGIDPSIILDVLGSAAAGAEAVGSG
*Methylibium petroleiphilum*	Mpe_A1879	4E-40	Mpe_A1878	3E-79	X	Mpe_A1877	Large RTX protein	QALNEGDPDAATAAGGPASGGEA
*Vibrio parahaemolyticus*	VPA1734	9E-40	VPA1735	3E-60	X	VPA0668	TP1SS repeat 143	QQAILEGADPTAILEATAAGGDASG

Given its strict conservation and peculiar conformation, we targeted W^125^ in a site-directed mutagenesis study, replacing its side chain non-conservatively with a glutamate residue. The mutant output domain expressed and purified indistinguishably from the wild-type protein but had distinct functional properties. In a purified system using hexahistidine (His_6_)–tagged LapG, a periplasmic cysteine protease that binds to LapD's output domain in a c-di-GMP-dependent manner (see Newell et al. [29]), we could efficiently pull down the untagged wild-type output domain (Figure 10D). Luminescent detection-based quantification indicates a binding stoichiometry of two LapG molecules per output domain dimer at saturating conditions. This result indicates that in the absence of the transmembrane and cytoplasmic domains, the output domain adopts a LapG-binding-competent state. In contrast, the output domain mutant W^125^E failed to interact with LapG in this assay. Consistent with these results, a full-length allele harboring the W^125^E mutation failed to restore LapD-dependent biofilm formation in a Δ*lapD* genetic background (Figure 10E). The periplasmic loss-of-function mutation is also dominant over the highly activating S^229^D mutation when introduced in the same allele, underlining the functional importance of W^125^ in transmitting cytosolic signaling events to the periplasm.

### Structure-Based Model for the Regulation of Periplasmic Proteases in Bacteria

**Figure 11:**
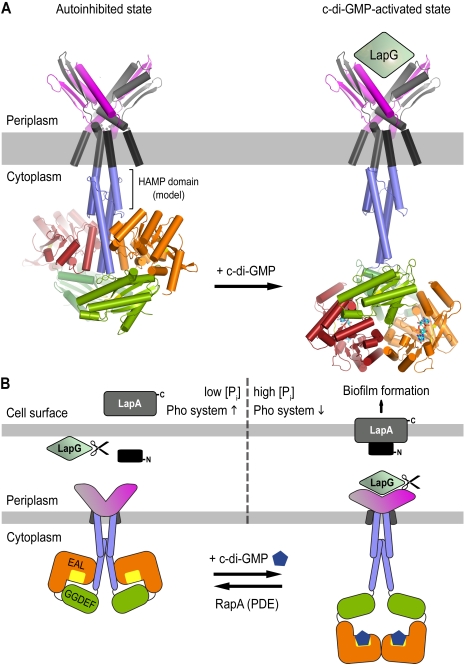
Structure-based model for LapD inhibition and activation. (A) Structural model of full-length LapD. We derived models for the autoinhibited and activated, c-di-GMP-bound state of LapD based on the crystal structures described here. Only the c-di-GMP-bound receptor is capable of LapG binding in the periplasm. The HAMP domains were modeled based on sequence alignments and available structural information [49],[50]. (B) Model for LapD-mediated control of biofilm formation. The cartoon presents the current model for biofilm formation controlled by the c-di-GMP receptor LapD, based on our structural and functional analyses, previous results [24]–[26], and the companion paper by Newell et al. [29].

Our structural analyses of LapD revealed an autoinhibited conformation of the cytosolic domains in the absence of c-di-GMP, a dimeric state of c-di-GMP-bound EAL domains in the active state, and a domain-swapped dimer of the periplasmic output domain that is competent for LapG binding. The HAMP domain was modeled based on available structural information for this relay module, with the S helix forming a continuous extension of the HAMP domain's second helix [49],[50]. In conjunction with the biochemical and genetic analyses described in an accompanying manuscript, we propose the following model for the activation of LapD and its mechanism of inside-out signaling across the inner bacterial membrane (Figure 11). The S helix and GGDEF domain function as a physical lock, gating access of c-di-GMP to the EAL domain. In this conformation, LapD's output domain is held in a LapG-binding-incompetent state, and hence LapG gains access to and cleaves LapA, releasing this critical biofilm adhesin from the cell surface. An increase in the cellular c-di-GMP level, concomitant with a sampling of a c-di-GMP-binding-competent conformation of LapD, will outcompete the inhibitory interactions in the cytoplasmic domains, likely accompanied by a large conformational change allowing EAL domain dimerization. Coupling between dimerization and c-di-GMP binding may further contribute to the efficiency of the activation switch, by preventing reversal to the autoinhibited state. Many mutations in the cytoplasmic module including the HAMP domain lead to aberrant, constitutive activation of LapD (Figures 8 and 9) [24]. These data suggest that intrinsic autoinhibitory interactions are indeed necessary to prevent the system from adopting a constitutively active conformation.

Based on the primary sequence and secondary structure predictions, the HAMP domain is directly linked to the GGDEF–EAL domain module via the S helix. HAMP domains occur in a large number of predominantly transmembrane sensor proteins that transmit signals from the environment across the cell membrane to elicit an intracellular response (outside-in signaling) [21]. Rotation of the helices in HAMP dimers has been described as the main mechanism for signal transmission [49]. It is conceivable that the EAL domain–S helix interaction stabilizes the off state, and that the release of the EAL domain from the S helix will allow the receptor to relax. The disengagement may trigger a rotation in the HAMP domain in a similar fashion to in other HAMP domains [49],[50], yielding a conformational change in the output domain and allowing the periplasmic domain of LapD to sequester LapG.

What is the relevance of the unusual fold of LapD's output domain? Unlike CitA and related sensor proteins, which bind small molecules in the periplasm and relay this information to the inside of the cell, LapD sequesters a periplasmic protein upon receiving a cytosolic signal. We speculate that a domain-swapped fold would respond more efficiently and precisely in coupling conformational changes in the cytosolic domains across the membrane than canonical dimeric periplasmic domains. One may consider the periplasmic domain of LapD as a single domain given the extensive sharing of structural elements and a negligible monomer–dimer transition. Given the functional importance and the particular position of W^125^, we hypothesize that the output domain may act as a molecular ruler, with the tryptophan residues forming the tips of a caliper. Varying the angle between the arms of the V-shaped fold upon c-di-GMP-triggered HAMP domain rotation could form the basis for modulating binding of LapG in the periplasm, assuming that both tryptophan residues of the dimeric, periplasmic fold interact with LapG (monomers or dimers).

Although competent for specific LapG binding, the isolated LapD output domain failed to compete for LapG sequestration with the full-length c-di-GMP-bound receptor (P. D. N., unpublished data). It is likely that the intracellular and transmembrane domains facilitate the formation of a stable, high-affinity state. In addition, removal of the domain from its native context may alter its conformation. The observation that the isolated output domain can bind LapG is consistent with a model in which the dinucleotide-free, intracellular domains hold the receptor in an autoinhibited conformation that relaxes into a LapG-binding state upon activation. Consequently, deletion of the regulatory domains would allow for the output domain to adopt the active, LapG-binding conformation. In addition, potential higher-order oligomerization of LapD into lattices may contribute to sequestering LapG over larger membrane surfaces and to the fine-tuning of the signaling system. Two crystal structures described here, of the output domain and the c-di-GMP-bound EAL domain, show some potentially relevant higher-order interactions (Figure S7C and S7D). Further experiments will be required to determine the oligomeric state of full-length LapD in the absence and presence of c-di-GMP.

### Conservation of Signaling Systems Involving LapD Homologs

Based on sequence conservation, LapD homologs in other *Pseudomonas* strains, including *P. putida* and *P. aeruginosa*, are likely to function in a similar fashion (Figure S1; Table 2) [24],[27]. While LapD and LapG from *P. aeruginosa* (PA1433 and PA1434, respectively) show a high degree of sequence conservation and functionally rescue deletions in these genes in *P. fluorescens*, no biofilm phenotype has been associated with this signaling system in their native strain [23], consistent with the absence of an obvious LapA homolog in this species. In contrast, we identified similar effector systems and targets in more distant genera including *Legionella* and various *Vibrio* strains. In all these bacteria, *lapD* and *lapG* homologs with conserved, functionally important residues exist within the same operon (Figure S1; Table 2). LapD from *V. cholerae El Tor* represents a special case since its EAL domain is encoded by a second gene, separated from the transmembrane receptor containing the output, HAMP, and GGDEF domains. While the relevance of this finding requires further investigation, these genes have been found upregulated in rugose strains of *V. cholerae*, associated with increased biofilm formation [51].

The bioinformatic analysis also detected the presence of associated ABC transporters in genomes encoding LapD homologs, as in the case of *P. fluorescens*. Putative substrates of the cysteine protease LapG may fall into one of two categories. Newell et al. [29] identified the large adhesin LapA as a LapG substrate, involved in biofilm formation and stability in *P. fluorescens*. Based on the cleavage site sequence, other LapA homologs were identified in a variety of strains. In addition, we predict that LapG homologs may have different substrates in systems for which no clear LapA-type proteins could be identified. Regions with homology to the LapG-cleavage site of LapA have been identified in RTX-like bacterial toxins, and for the majority of such candidate substrates, these proteins are encoded in close genetic proximity to *lapD* and *lapG* homologs.

The GGDEF–EAL domain–containing proteins described here are degenerate with respect to their active sites, lack catalytic activity, and function as c-di-GMP receptors. A similar system has been previously described in *Escherichia coli*. Unlike LapD, the transmembrane HAMP–GGDEF–EAL domain–containing protein CsrD regulates degradation of regulatory RNAs, but we speculate that the cytosolic module may be autoregulated in a similar fashion [52]. Other proteins containing the tandem domain module with a higher degree of conservation at the putative enzyme active sites exist in association with a HAMP domain in some bacterial genomes (e.g., *V. cholerae*). The mechanism described for LapD may also be applicable to these systems, in which the HAMP domain and S helix could be regulatory features to control the phosphodiesterase and/or diguanylate cyclase activity in the outside-in signaling mechanism, thus leading to changes in cellular c-di-GMP levels.

### Conclusions

Here, we elucidated the molecular mechanism underlying the function and regulation of *P. fluorescens* LapD, a transmembrane receptor essential for biofilm formation in this strain. Similar receptors are conserved in many bacteria where they control a LapG-type, periplasmic protease. LapD is autoinhibited with regard to c-di-GMP binding by interactions of the EAL domain with the S helix and the GGDEF domain. Receptor activation requires the concurrent release of the EAL domain from these interactions and the binding of c-di-GMP, which triggers a conformational change in the output domain from an incompetent to a competent state with regard to LapG binding [29]. Mutations in the regulatory features that weaken the autoinhibitory interactions render LapD constitutively active even under phosphate starvation (low c-di-GMP levels; Figure 9). This is in contrast to other c-di-GMP receptors with known structure, such as PilZ domain–containing proteins [53],[54], VpsT [14], and the GGDEF–EAL domain–containing protein FimX [36]. In all these cases, the c-di-GMP-binding site appears to be readily accessible in the apo states (Figure S6). In PlzD, dinucleotide binding introduces a conformational change that changes the relative orientation of its two domains [53]. In FimX, the EAL domains form the distal tips of an elongated, dimeric protein [36]. c-di-GMP binding to the isolated EAL domain or the full-length protein is indistinguishable, and no major conformational change has been observed for FimX upon dinucleotide binding, suggesting a mode of signal transmission that may rely on partner proteins [36],[55].

Given the occurrence of the HAMP–GGDEF–EAL domain module in many other proteins from different free-living and pathogenic bacterial species, the results discussed here will have broad implications for receptors predicted to mediate either inside-out or outside-in signaling involving the bacterial second messenger c-di-GMP.

## Materials and Methods

### Protein Expression, Purification, and Crystallography

The dual GGDEF–EAL domain module (LapD^dual^; residues 220–648), the EAL domain (LapD^EAL^; residues 399–648), and the periplasmic output domain (LapD^output^; residues 22–151) of *P. fluorescens* Pf0-1 LapD were produced following standard molecular biology and liquid chromatography techniques. Crystals were obtained by hanging drop vapor diffusion, and datasets were collected using synchrotron radiation at the Cornell High Energy Synchrotron Source (Ithaca, New York). Detailed protocols are provided in Text S1.

### Size Exclusion Chromatography–Coupled Static MALS

For MALS measurements, purified proteins (20–320 µM, injected concentration) were subjected to size exclusion chromatography (SEC) using a WTC-030S5 (for LapD^dual^) or WTC-015S5 (for LapD^EAL^) column (Wyatt Technology) equilibrated in gel filtration buffer (25 mM Tris-HCl [pH 8.4] and 250 mM NaCl). Where specified, wild-type or mutant LapD protein variants were incubated with c-di-GMP (500 µM), produced enzymatically (see Text S1), for 30 min at room temperature prior to SEC. The SEC system was coupled to an 18-angle static light scattering detector and a refractive index detector (DAWN HELEOS-II and Optilab T-rEX, respectively, Wyatt Technology). Data were collected at 25°C every second at a flow rate of 1.0 ml/min and analyzed with the software ASTRA, yielding the molecular weight and mass distribution (polydispersity) of the samples. For data quality control and normalization of the light scattering detectors, monomeric bovine serum albumin (Sigma) was used.

### Sedimentation Equilibrium Analysis

Ultracentrifugation experiments were performed at 20°C in a Beckman ProteomeLab XL-A centrifuge equipped with an AN-60 rotor and absorbance optics. Sedimentation equilibrium data were recorded for 12–15 h each at speeds of 10,000, 14,000, and 20,000 rpm. Scans were taken at 1-h intervals with a 0.001-cm step size along the radial axis and five replicates per data point. Attainment of sedimentation equilibrium was verified using the program WinMATCH (D. A. Yphantis and J. W. Lary; www.biotech.uconn.edu/auf). Six-sector cells were loaded with 1×, 2×, and 4× dilutions of ∼12 µM stock solutions of either wild-type or S^229^D LapD^dual^ in 25 mM Tris (pH 7.5) and 150 mM NaCl, either neat or supplemented to a final concentration of 20 µM c-di-GMP. Curves collected at all three speeds for all three channels were globally fit. Protein partial specific volume (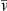) and buffer density and viscosity (ρ,η) were calculated using the program SEDNTERP [56]. Sedimentation equilibrium data were analyzed using the program SEDANAL [57], using either single-species models or models including dinucleotide binding and protein dimerization.

### Semi-Quantitative c-di-GMP Binding Assays

Proteins (250 µM) were preincubated with excess c-di-GMP (500 µM) at 4°C and separated from unbound dinucleotide via SEC. SEC-eluted protein peaks were collected, concentrated to a final concentration of 200 µM to normalize for protein content, heat-denatured, and filtered through Microcon Centrifugal Filter Units (Millipore, 10 kDa cutoff). Dinucleotide content in the resulting samples was analyzed on a C18 reverse-phase HPLC column by using a methanol-phosphate gradient (buffer A: 100 mM monobasic potassium phosphate [pH 6.0]; buffer B: 70% buffer A, 30% methanol) [18]. Purified nucleotides were used for standardization. Integrated areas of the c-di-GMP peaks from three independent experiments were plotted relative to those for the wild-type LapD^dual^ and LapD^EAL^ protein constructs.

Binding of [^32^P]-c-di-GMP to purified LapD^dual^ or LapD^EAL^ (1 µM) was assessed by filter binding assays as described before [24],[25]. Unspecific background binding was determined by using bovine serum albumin, and was subtracted from the data obtained for LapD-containing samples. Data were fitted to a one-site-specific binding model *Y* = *B*_max_·*X*/(*K*_d_ + *X*) in GraphPad Prism (*B*_max_, maximum specific binding; *K*_d_, apparent binding constant).

### Protein Pull-Down Assay

His_6_-tagged LapG was incubated with NiNTA superflow resin (Qiagen) in low-salt binding buffer (25 mM Tris-HCl [pH 8.4], 75 mM NaCl, 25 mM KCl, and 40 mM Imidazole). After removal of any unbound protein in consecutive wash steps, untagged LapD output domain variants were added to the reaction and incubated for 1 h at 4°C under nutation. The resin was extensively washed in low-salt binding buffer. The remaining affinity-bound proteins or protein complexes were eluted from the slurry in elution buffer (25 mM Tris-HCl [pH 8.4], 500 mM NaCl, and 300 mM Imidazole) and visualized using standard denaturing gel electrophoresis (SDS-PAGE). For quantification, gels were stained with SYPRO Ruby gel stain (Molecular Probes) following the manufacturer's directions, and imaged on a VersaDoc MP system (Bio-Rad).

### Strains and Growth Conditions

Routine culturing of *P. fluorescens* Pf0-1 and *E. coli* was done in lysogeny broth at 30°C and 37°C, respectively. When appropriate, antibiotics were added to the medium at the following concentrations: *E. coli*, 10 µg/ml gentamicin; *P. fluorescens*, 20 µg/ml gentamicin. Plasmids were introduced into *P. fluorescens* by electroporation as described previously [58]. K10T medium for biofilm assays was prepared as described previously [59]. K10T-π is 50 mM Tris-HCl (pH 7.4), 0.2% (wt/vol) Bacto tryptone, 0.15% (vol/vol) glycerol, and 0.61 mM Mg_2_SO_4_. K10T-1 medium is K10T-π amended with 1 mM K_2_HPO_4_. A list of strains and plasmids used in the cell-based assays is provided in Table S2.

### Quantitative Biofilm Formation and Surface Attachment Assays

To quantify biofilm formation, strains were grown statically for 6 h in K10T-1 medium as described previously [24]. Biofilm biomass was stained with 0.1% crystal violet for 15 min, the stain was dissolved, and the biofilm quantified by spectrophotometry, measuring the optical density at 550 nm. We analyzed the effects of inorganic phosphate starvation on attachment by comparing biofilm levels in high-phosphate (K10T-1) and low-phosphate (K10T-π) media over time, as done previously [24].

### Assessment of LapD Protein Levels by Western Blot

LapD proteins expressed in *P. fluorescens* Pf0-1 were visualized by Western blot as described previously [24], with the following modifications. Blots were probed for the His_6_ epitope with a rabbit anti-His_6_ antibody (Genscript). Samples consisted of clarified cell lysates prepared by harvesting cells from 3 ml of overnight culture, sonicating 3×10s in 500 µl of buffer (20 mM Tris [pH 8] and 10 mM MgCl_2_), and pelleting debris at 15,000*g* for 12 min. Samples were normalized to protein concentration using the BCA kit (Pierce).

### Accession Numbers

Atomic coordinates and structure factors have been deposited in the RCSB Protein Data Bank (http://www.pdb.org) under the ID codes 3pjt, 3pju, 3pjv, 3pjw, and 3pjx.

## Supporting Information

Figure S1 **Sequence alignment of LapD homologs.** A sequence alignment of LapD homologs from various species was generated with ClustalW2 [60] and formatted with ESPript [61]. Key residues discussed in the manuscript are marked with closed green arrows. The degenerate GGDEF and EAL signature motifs (RGGEF and KVL, respectively) are marked with yellow bars. Secondary structure elements are shown based on the crystallographic data and secondary structure predictions for the transmembrane and HAMP domains. The following sequences were used to generate the alignment: *P. fluorescens* Pf0-1 (LapD, YP_345864), *P. putida* KT2440 (NP_742334), *P. aeruginosa* PA01 (NP_250124), *Pectobacterium carotovorum* subsp. *brasiliensis* PBR1692 (ZP_03826388), *Citrobacter* sp. ATCC 29220 (ZP_06355256), *Polaromonas* sp. JS666 (YP_547171), *Rhodoferax ferrireducens* T118 (YP_524995), *Dechloromonas aromatica* RCB (YP_286553), *Cellvibrio japonicus* Ueda107 (YP_001981887), *L. pneumophila* str. *Lens* (YP_126219), *Geobacter* sp. M18 (ZP_05313414), *V. alginolyticus* 12G01 (ZP_01258281), *V. parahaemolyticus* AQ3810 (ZP_01990882), *V. harveyi* HY01 (ZP_01986262), *V. shilonii* AK1 (ZP_01866121), *V. cholerae* 1587 (ZP_01950486), *V. fischeri* ES114 (YP_207124), and *V. angustum* S14 (ZP_01233947).
(4.74 MB PDF)

Figure S2 **Crystal forms of LapD^dual^ and LapD^EAL^•c-di-GMP.** (A) LapD^dual^. Two independent crystal forms were obtained for LapD^dual^. The resulting structures were superimposed on the EAL domain and shown as protein backbone traces. (B) c-di-GMP-bound LapD^EAL^. Two independent crystal forms were obtained for LapD^EAL^. Both crystal lattices show the same dimeric assembly of EAL domains. Dimers were superimposed on one EAL domain and shown as protein backbone traces. (C) Stereo views. Stereo views of the structural comparisons shown in (A) and (B) are shown. In this view, the EAL domains of LapD^dual^ and LapD^EAL^•c-di-GMP are shown in a similar orientation.
(7.01 MB TIF)

Figure S3 **Comparison of the GGDEF domains from LapD and PleD.** (A) Sequence alignment. Sequences of GGDEF domains with known structure were used to generate the alignment [17],[18],[36]. Conserved residues involved in nucleotide binding and hydrolysis are marked with asterisks [17],[38]. The GGDEF motif is highlighted with a yellow bar. (B) Overview. Structures of GGDEF domains of LapD and PleD (PDB ID 2v0n) are shown as a ribbon presentation [38]. A GTP analog bound to the active site of PleD is shown as a stick presentation. (C) GTP binding site. A close-up view of the active site is shown. Residues that in PleD are involved in nucleotide and divalent cation coordination are shown as a stick presentation. Left labels correspond to the LapD sequence; right labels correspond to the PleD sequence.
(1.79 MB TIF)

Figure S4 **Comparison of c-di-GMP-bound LapD^EAL^ and YkuI dimers.** (A) Sequence alignment. Sequences of EAL domains with known structure were used to generate the alignment [34]–[37],[39]. Conserved residues in active phosphodiesterases are marked with asterisks [37]. The EAL motif is highlighted with a yellow bar. The loop and helix involved in dimerization are marked with a green and orange bar, respectively. (B) Overview. Structures of EAL domain dimers of LapD and YkuI bound to c-di-GMP (PDB ID 2w27) are shown as a ribbon presentation [35]. c-di-GMP is shown as a stick presentation. Structures were superimposed on one of the EAL domains of the dimeric assemblies. (C) c-di-GMP-binding site. A close-up view of the nucleotide-binding pocket is shown. Residues involved in c-di-GMP (and, in the case of YkuI, divalent cation) coordination are shown as a stick presentation. Left labels correspond to the LapD sequence; right labels correspond to the YkuI sequence.
(7.34 MB TIF)

Figure S5 **GGDEF–EAL domain interactions and S helix–GGDEF domain linker conformation observed in apo-LapD^dual^.** (A) GGDEF–EAL domain interaction. Close-up views are shown for regions of direct contact between the GGDEF and EAL domains in the autoinhibited structure of LapD^dual^. The GGDEF and EAL domains are colored in green and orange, respectively. The S helix is colored in blue. (B) Nucleotide-binding pocket in apo-LapD^dual^. A close-up view of the c-di-GMP-binding pocket of LapD is shown (right panel). c-di-GMP is shown as a stick presentation after superimposing the crystal structure of LapD^EAL^•c-di-GMP onto the EAL domain of apo-LapD^dual^. The interacting residue pair R^450^/E^262^ in LapD is incompatible with c-di-GMP binding. The left panels show surface presentations of apo-LapD^dual^. The middle panel shows accessibility of the c-di-GMP-binding site, with c-di-GMP taken from LapD^EAL^•c-di-GMP after superimposition. (C) S helix–GGDEF connector. The S helix and the GGDEF domain are connected via a short loop that forms a tight turn. The loop conformation is conserved in other GGDEF domain–containing proteins, and is stabilized by the interaction between two residues D^239^ and R^316^, which are strictly conserved in many GGDEF domain–containing proteins [17],[18],[38],[41]. The arginine residue is directly preceding the GGDEF domain signature motif (GGDEF or GGEEF in active cyclases; RGGEF in LapD); the aspartate residue is located at the N-terminus of the loop. Its strict sequence and conformational conservation suggest a functional importance of the connector loop, likely restricting the conformational freedom between adjacent domains.
(9.22 MB TIF)

Figure S6 **Structural comparion of LapD with other c-di-GMP receptors.** (A) LapD. The monomoric apo-LapD^dual^ structure is shown as a surface presentation (top). The middle panel shows the c-di-GMP-bound EAL domain of LapD in two orthogonal views. The dinucleotide-binding site is colored in red. The conformation of c-di-GMP is similar to that observed in other EAL domains such as FimX, YkuI, and BlrP1 (bottom panel) (see also Figure S4) [17],[18],[34]–[38]. (B) VpsT (PDB IDs 3kln and 3klo). The transcription factor VpsT from *V. cholerae* exists in a monomer–dimer equilibrium. An apo-VpsT monomer is shown as a surface presentation (top panel). The dimeric species is stabilized by c-di-GMP binding to the base of the regulatory receiver domain (middle panel) [14]. Two molecules of c-di-GMP form an intercalated dimer, similar to the binding mode observed for the inhibitory site binding in active diguanylate cyclases [17],[38]. The dinucleotide binding site is shown in red. (C) PilZ domains (PDB IDs 1yln, 2rde, 3yg, and 3kyf). The PliZ domain–containing protein PlzD/VCA0042 forms homodimers via its YcgR-N* domain. The PilZ domains form separate lobes of the protein. PilZ domain–containing proteins have been shown to bind either one or two mutually intercalated molecules of c-di-GMP [53],[54]. The dinucleotide-binding site is shown in red.
(7.57 MB TIF)

Figure S7 **Structural analysis of LapD^output^ and potential mechanisms for higher-order oligomerization of LapD.** (A) Comparison between LapD^output^ protomers. The periplasmic output domain of LapD crystallized with two molecules in the asymmetric unit. The protomers were superimposed on the first two helices of the fold, revealing a minor, rigid-body rotation of one half of the molecule relative to the other half between the two protomers. The rotation occurs at the connecting loop between β2 and α3 that forms the crossing-over point in the domain-swapped dimer. (B) LapD^output^ crystal packing. Domain-swapped dimers of the output domain interact predominantly via two interfaces in the crystal lattices. One involves bottom-to-bottom interaction between LapD^output^ dimers via a conserved, hydrophobic patch coinciding with the putative membrane-interaction surface. The other interface involves hydrophobic interactions between the arms of the V-shaped output domain dimers. (C) Potential higher-order oligomerization based on the structure of LapD^output^. Crystal lattice contacts reveal a potential mode for higher-order assemblies of LapD. The close-up view (right panel) shows the hydrophobic contacts between output domain dimers. (D) Potential higher-order oligomerization based on the structure of LapD^EAL^•c-di-GMP. In the *C*222_1_ crystal lattice, EAL domains form higher-order lattices that may highlight a mode for receptor oligomerization in the membrane.
(7.15 MB TIF)

Figure S8 **Surface conservation and hydrophobicity of LapD^output^.** (A) Surface conservation. Based on an alignment of 18 sequences of LapD homologs (Figure S1), the sequence conservation was mapped onto the solvent-accessible surface of the output domain. One protomer is shown as a surface presentation, the other is shown as a ribbon presentation. Conserved motifs and individual residues are highlighted. Two views, separated by a 180° rotation, are shown. (B) Hydrophobicity mapped onto the molecular surface of LapD^output^. The surface is colored according to the hydrophobicity of accessible residues. Hydrophobic residues are shown in green; polar and charged residues are in gray and pink, respectively.
(3.66 MB TIF)

Table S1 **X-ray data collection and refinement statistics.**
(0.08 MB DOC)

Table S2 **Strains and plasmids.**
(0.05 MB DOC)

Text S1 **Supporting information.** The file includes supplemental Materials and Methods and associated references.
(0.07 MB DOC)

## Abbreviations

c-di-GMP: bis-(3′–5′) cyclic dimeric guanosine monophosphate
GTP: guanosine triphosphate
His_6_: hexahistidine
MALS: multi-angle light scattering
PDB: RCSB Protein Data Bank
rmsd: root mean square deviation
S helix: signaling helix
SD: standard deviation
SEC: size exclusion chromatography
