# Supplementary material for: Structural Basis for c-di-GMP-Mediated Inside-Out Signaling Controlling Periplasmic Proteolysis
Source: PLoS Biol. 2011 Feb 1;9(2):e1000588. doi: 10.1371/journal.pbio.1000588 (PMC3032553; doi:10.1371/journal.pbio.1000588)
Supplement: Figure S1 — Sequence alignment of LapD homologs. A sequence alignment of LapD homologs from various species was generated with ClustalW2 [60] and formatted with ESPript [61]. Key residues discussed in the manuscript are marked with closed green arrows. The degenerate GGDEF and EAL signature motifs (RGGEF and KVL, respectively) are marked with yellow bars. Secondary structure elements are shown based on the crystallographic data and secondary structure predictions for the transmembrane and HAMP domains. The following sequences were used to generate the alignment: P. fluorescens Pf0-1 (LapD, YP_345864), P. putida KT2440 (NP_742334), P. aeruginosa PA01 (NP_250124), Pectobacterium carotovorum subsp. brasiliensis PBR1692 (ZP_03826388), Citrobacter sp. ATCC 29220 (ZP_06355256), Polaromonas sp. JS666 (YP_547171), Rhodoferax ferrireducens T118 (YP_524995), Dechloromonas aromatica RCB (YP_286553), Cellvibrio japonicus Ueda107 (YP_001981887), L. pneumophila str. Lens (YP_126219), Geobacter sp. M18 (ZP_05313414), V. alginolyticus 12G01 (ZP_01258281), V. parahaemolyticus AQ3810 (ZP_01990882), V. harveyi HY01 (ZP_01986262), V. shilonii AK1 (ZP_01866121), V. cholerae 1587 (ZP_01950486), V. fischeri ES114 (YP_207124), and V. angustum S14 (ZP_01233947). (4.74 MB PDF) [file pbio.1000588.s001.pdf]

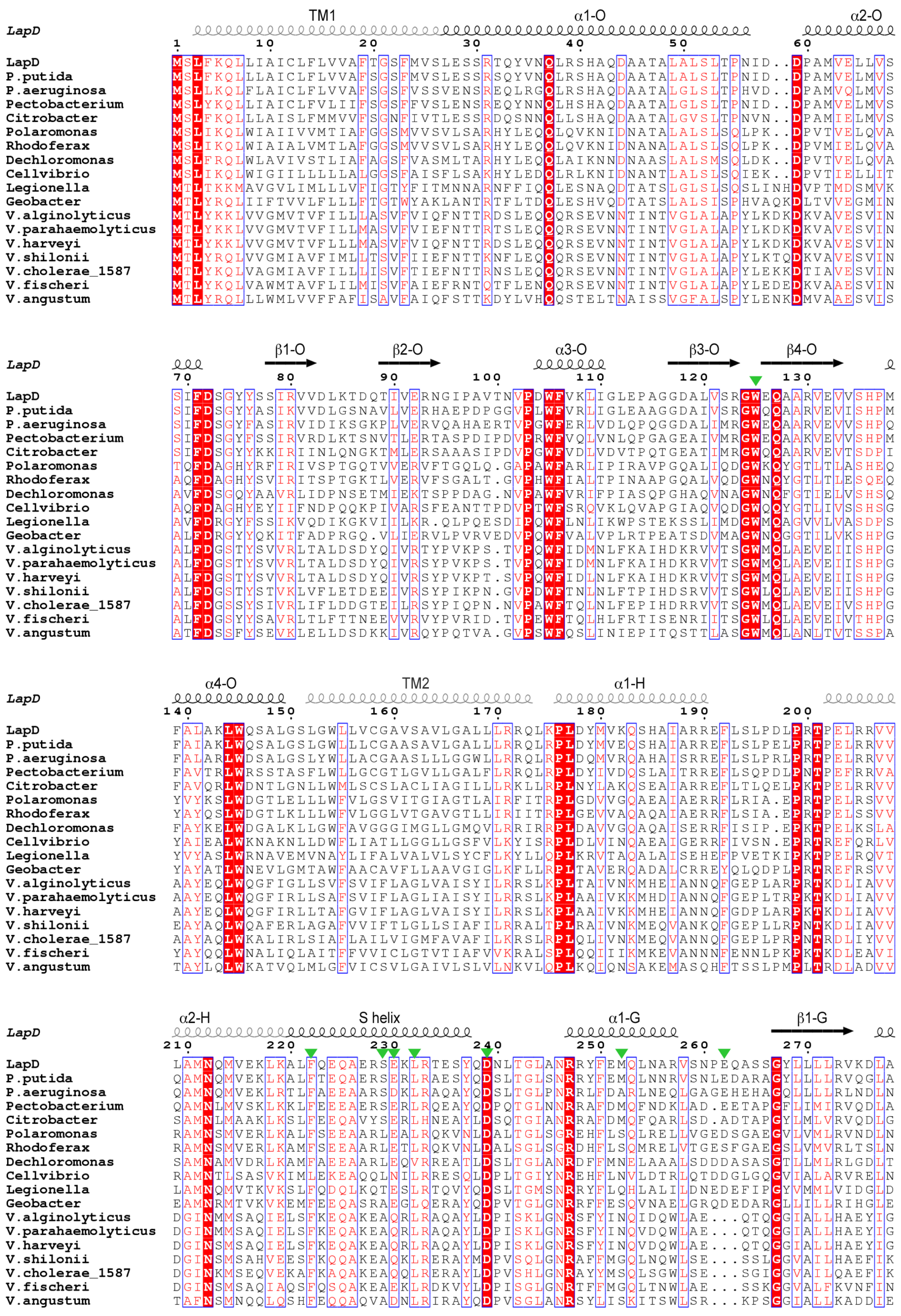

LapD
TM1
α1-O
α2-O
β1-O
β2-O
α3-O
β3-O
β4-O
α4-O
TM2
α1-H
α2-H
S helix
α1-G
β1-G
LapD
P.putida
P.aeruginosa
Pectobacterium
Citrobacter
Polaromonas
Rhodoferax
Dechloromonas
Cellvibrio
Legionella
Geobacter
V.alginolyticus
V.parahaemolyticus
V.harveyi
V.shilonii
V.cholerae_1587
V.fischeri
V.angustum

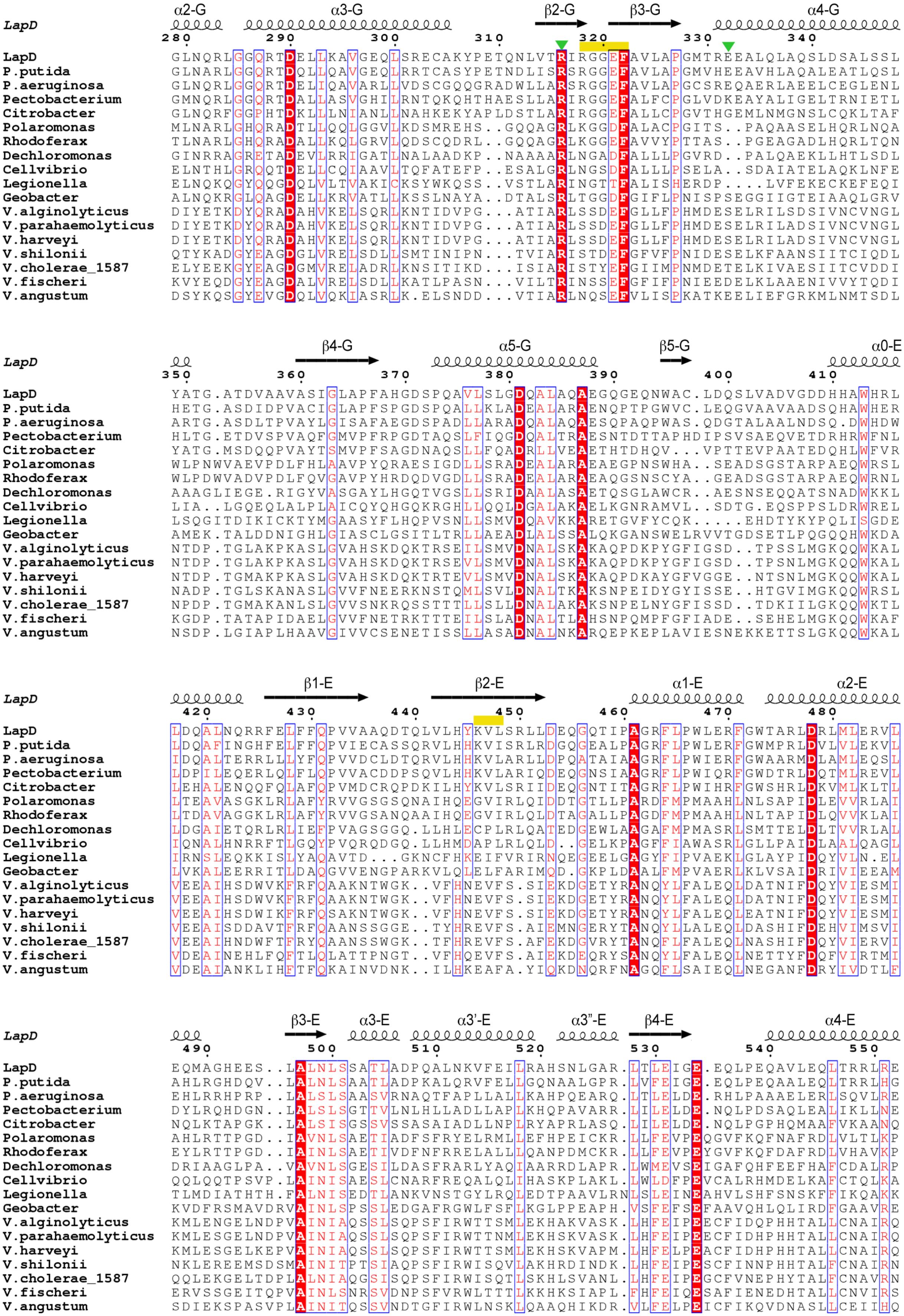
α2-G α3-G β2-G β3-G α4-G
LapD
280 290 300 310 320 330 340
LapD GLNQRLGGQRTDELLKAVGEQLSRECAKYPETQNLVTRIRGGEFAVLAPGMTREEALQLAQSLDSALSSL
P.putida GLNARLGGQRTDQLLQAVGEQLRRTCASYPETNDLISRSRGGEFAVLAPGMVHEEAVHLAQALEATLQSL
P.aeruginosa GLNQRLGGQRTDELIQAVARLLVDSCGQQGRADWLLARSRGGEFAVLAPGCSREQAERLAEELCEGLENL
Pectobacterium GMNQRLGGQRTDALLASVGHILRNTQKQHTHAESLLARIRGGEFALFCPGLVDKEAYALIGELTRNIETL
Citrobacter GLNQRFGGPHTDKLLLNIANLLNAHKEKYAPLDSTLARIRGGEFALLCPGVTHGEMLNMGNSLCQKLTAF
Polaromonas MLNARLGHQRADTLLQQLGGVLKDSMREHS..GQQAGRLKGGDFALACPGITS..PAQAASELHQRLNQA
Rhodoferax TLNARLGHQRADALLKQLGRVLQDSCQDRL..GQQAGRLKGGEFAVVYPTTAS..PGEAGADLHQRLTQN
Dechloromonas GINRRAGRETADEVLRRIGATLNALAADKP..NAAAARLNGADFALLLPGVRD..PALQAEKLLHTLSDL
Cellvibrio ELNTHLGRQQTDELLCQIAAVLTQFATEFP..ESALGRLNGSDFALLLPSELA..SDAIATELAQKLNFE
Legionella ELNQKQGYQQGDQLVLTVAKICKSYWKQSS..VSTLARINGTTFALISHERDP....LVFEKECKEFEQI
Geobacter ALNQKRGLQAGDELLKRVATLLKSSLNAYA..DTALSRLTGGDFGIFLPNISPSEGGIIGTEIAAQLGRV
V.alginolyticus DIYETKDYQRADAHVKELSQRLKNTIDVPG...ATIARLSSDEFGLLFPHMDESELRILSDSIVNCVNGL
V.parahaemolyticus DIYETKDYQRADAHVKELSQRLKNTIDVPG...ATIARLSSDEFGLLFPHMDESELRILADSIVNCVNGL
V.harveyi DIYETKDYQRADAHVKELSQRLKNTIDVPG...ATIARLSSDEFGLLFPHMDESELRILADSIVNCVNGL
V.shilonii QTYKADGYEAGDGLVRELSDLLSMTINIPN...TVIARISTDEFGFVFPNIDESELKFAANSIITCIQDL
V.cholerae_1587 ELYEEKGYEAGDGMVRELADRLKNSITIKD...ISIARISTYEFGIIMPNMDETELKIVAESIITCVDDI
V.fischeri KVYEQDGYEAGDALVRELSDLLKATLPASN...VILTRINSSEFGFIFPNIEEDELKLAAENIVVYTQDI
V.angustum DSYKQSGYEVGDQLVQKIASRLK.ELSNDD...VTIARLNQSEFVLISPKATKEELIEFGRKMLNMTSDL
β4-G α5-G β5-G α0-E
LapD
350 360 370 380 390 400 410
LapD YATG.ATDVAAVASIGLAPFAHGDSPQAVLSLGDQALAQAEGQGEQNWAC.LDQSLVADVGDDHHAWHRL
P.putida HETG.ASDIDPVACIGLAPFSPGDSPQALLKLADEALARAENQPTPGWVC.LEQGVAAVAADSQHAWHER
P.aeruginosa ARTG.ASDLTPVAYLGISAFAEGDSPADLLARADQALAQAESQPAQPWAS.QDGTALAALNDSQ.DWHDW
Pectobacterium HLTG.ETDVSPVAQFGMVPFRPGDTAQSLFIQGDQALTRAESNTDTTAPHDIPSVSAEQVETDRHRWFNL
Citrobacter YATG.MSDQQPVAYTSMVPFSAGDNAQSLLFQADRLLVEAETHTDHQV...VPTTEVPAATEDQHLWFVR
Polaromonas WLPNWVAEVPDLFHLAAVPYQRAESIGDLLSRADEALARAEAEGPNSWHA..SEADSGSTARPAEQWRSL
Rhodoferax WLPDWVADVPDLFQVGAVPYHRDQDVGDLLSRADEALARAEAQGSNSCYA..GEADSGSTARPAEQWRNL
Dechloromonas AAAGLIEGE.RIGYVASGAYLHGQTVGSLLSRIDAALASAETQSGLAWCR..AESNSEQQATSNADWKKL
Cellvibrio LIA..LGQEQLALPLAICQYQHGQKRGHLLQQLDGALAKAELKGNRAMVL..SDTG.EQSPPSLDRWREL
Legionella LSQGITDIKICKTYMGAASYFLHQPVSNLLSMVDQAVKKARETGVFYCQK.....EHDTYKYPQLISGDE
Geobacter AMEK.TALDDNIGHLGIASCLGSITLTRLLAEADLALSSALQKGANSWELRVVTGDSETLPQGQQHWKDA
V.alginolyticus NTDP.TGLAKPKASLGVAHSKDQKTRSEILSMVDNALSKAKAQPDKPYGFIGSD..TPSSLMGKQQWKAL
V.parahaemolyticus NTDP.TGLAKPKASLGVAHSKDQKTRSEVLSMVDNALSKAKAQPDKPYGFIGSD..TPSNLMGKQQWKAL
V.harveyi NTDP.TGMAKPKASLGVAHSKDQKTRTEVLSMVDNALSKAKAQPDKAYGFVGGE..NTSNLMGKQQWKAL
V.shilonii NADP.TGLSKANASLGVVFNEERKNSTQMLSVLDNALTKAKSNPEIDYGYISSE..HTGVIMGKQQWRSL
V.cholerae_1587 NPDP.TGMAKANLSLGVVSNKRQSSTTTLLSLLDNALAKAKSNPELNYGFISSD..TDKIILGKQQWKTL
V.fischeri KGDP.TATAPIDAELGVVFNETRKTTTEILSLADNALTLAHSNPQMPFGFIADE..SEHELMGKQQWKAF
V.angustum NSDP.LGIAPLHAAVGIVVCSENETISSLLASADNALNKARQEPKEPLAVIESNEKKETTSLGKQQWKAL
β1-E β2-E α1-E α2-E
LapD
420 430 440 450 460 470 480
LapD LDQALNQRRFELFFQPVVAAQDTQLVLHYKVLSRLLDEQGQTIPAGRFLPWLERFGWTARLDRLMLERVL
P.putida LDQAFINGHFELFFQPVIECASSQRVLHHKVISRLRDGQGEALPAGRFLPWLERFGWMPRLDVLVLEKVL
P.aeruginosa IDQALTERRLLLYFQPVVDCLDTQRVLHHKVLARLLDPQATAIAAGRFLPWIERFGWAARMDLAMLEQSL
Pectobacterium LDPILEQERLQLFLQPVVACDDPSQVLHHKVLARIQDEQGNSIAAGRFLPWIQRFGWDTRLDQTMLREVL
Citrobacter LEHALENQQFQLAFQPVMDCRQPDKILHYKVLSRIIDEQGNTITAGRFLPWIHRFGWSHRLDKVMLKLTL
Polaromonas LTEAVASGKLRLAFYRVVGSGSQNAIHQEGVIRLQIDDTGTLLPARDFMPMAAHLNLSAPIDLEVVRLAI
Rhodoferax LTDAVAGGKLRLAFYRVVGSANQAAIHQEGVIRLQLDDTAGALLPAGDFMPMAAHLNLTAPIDLQVVKLAI
Dechloromonas LDGAIETQRLRLIEFPVAGSGGQ.LLHLECPLRLQATEDGEWLAAGAFMPMASRLSMTTELDLTVVRLAL
Cellvibrio IQNALHNRRFTLGQYPVQRQDGQ.LLHMDAPLRLQLD..GELKPAGFFIAWASRLGLLPAIDLAVQAGL
Legionella IRNSLEQKKISLYAQAVTD...GKNCFHKEIFVRIRNQEGEELGAGYFIPVAEKLGLAYPIDQYVLN.EL
Geobacter LVKALEERRITLDAQGVVENGPARKVLQLELFARIMQD.GKPLDAALFMPVAERLKLVSAIDRIVIEEAM
V.alginolyticus VEEAIHSDWVKFRFQAAKNTWGK..VFHNEVFS.SIEKDGETYRANQYLFALEQLDATNIFDQYVIESMI
V.parahaemolyticus VEEAIHSDWVKFRFQAAKNTWGK..VFHNEVFS.SIEKDGETYRANQYLFALEQLDATNIFDQYVIESMI
V.harveyi VEEAIHSDWIKFRFQSAKNTWGK..VFHNEVFS.SIEKDGETYRANQYLFALEQLEATNIFDQYVIESMI
V.shilonii VEEAISDDAVTFRFQAANSSGGE..TYHREVFS.AIEMNGERYTANQYLLALEQLDASHIFDEHVIMSVI
V.cholerae_1587 VEEAIHNDWFTFRYQAANSSWGK..TFHREVFS.AFEKDGVRYTANQFLFALEQLNASHIFDQYVIERVI
V.fischeri VDEAINEHLFQFTLQLATTPNGT..VFHQEVFS.AIEKDEQRYSANQYLFALEQLNETTYFDQFVIRTMI
V.angustum VDEAIANKLIHFTFQKAINVDNK..ILHKEAFA.YIQKDNQRFNAGQFLSAIEQLNEGANFDRYIVDTLF
β3-E α3-E α3'-E α3"-E β4-E α4-E
LapD
490 500 510 520 530 540 550
LapD EQMAGHEES..LALNLSSATLADPQALNKVFEILRAHSNLGAR.LTLEIGE.EQLPEQAVLEQLTRRLRE
P.putida AHLRGHDQV..LALNLSAATLADPKALQRVFELLGQNAALGPR.LVFEIGE.EQLPEQAALEQLTRRLHG
P.aeruginosa EHLRRHPRP..LALSLSAASVRNAQTFAPLLALLKAHPQEARQ.LTLELDE.RHLPAAAELERLSQVLRE
Pectobacterium DYLRQHDGN..LALSLSGTTVLNLHLLADLLAPLKHQPAVARR.LILELDE.NQLPDSAQLEALIKLLNE
Citrobacter NQLKTAPGK..LALSISGSSVSSASAIADLLNPLRYAPRLASQ.LILELDE.NQLPGPHQMAAFVKAANQ
Polaromonas AHLRTTPGD..IAVNLSAETIADFSFRYELRMLLEFHPEICKR.LLFEVPEQGVFKQFNAFRDLVLTLKP
Rhodoferax EYLRTTPGD..IAINLSAETIVDFNFRRELALLLQANPDMCKR.LLFEVPEYGVFKQFDAFRDLVHAVKP
Dechloromonas DRIAAGLPA..VAVNLSGESILDASFRARLYAQIAARRDLAPR.LWMEVSEIGAFQHFEEFHAFCDALRP
Cellvibrio QQLQQTPSVP.LAINISAESLCNARFREQALQLIHASKPLAKL.LWLDFPEVCALRHMDELKAFCTQLKA
Legionella TLMDIATHTH.FALNISEDTLANKVNSTGYLRQLEDTPAAVLRNLSLEINEAHVLSHFSNSKFFIKQAKK
Geobacter KVDFRSMAVDRVAINLSPSSLEDGAFRGWLFSFLKGLPPEAPH.VSFEFSEFAAVQHLQLIRDFGAAVRE
V.alginolyticus KMLENGELNDPVAINIAQSSLSQPSFIRWTTSMLEKHAKVASK.LHFEIPEECFIDQPHHTALLCNAIRQ
V.parahaemolyticus KMLESGELNDPVAINIAQSSLSQPSFIRWTTNMLEKHSKVASK.LHFEIPEECFIDHPHHTALLCNAIRQ
V.harveyi KMLESGELKEPVAINIAQSSLSQPSFIRWTTSMLEKHSKVAPM.LHFELPEACFIDHPHHTALLCNAIRQ
V.shilonii NKLEREEMSDSMAINITPTSIAQPSFIRWISQVLAKHRDINDK.LHFEIPESCFINNPHHTALFCNAIRN
V.cholerae_1587 QQLEKGELTDPLALNIAQGSISQPSFIRWISQTLSKHLSVANL.LHFEIPEGCFVNEPHYTALFCNAVRN
V.fischeri ERVSSGEITQPVAINLSNRSVDNPSFVRWLTTTLKKHKSIAPL.FHFEIQESAFIENEDHTALLCNVIRS
V.angustum SDIEKSPASVPLAINITQSSVNDTGFIRWLNSKLQAAQHIKDR.VLFELPEICFIKQVDNASLLCEIIHQ

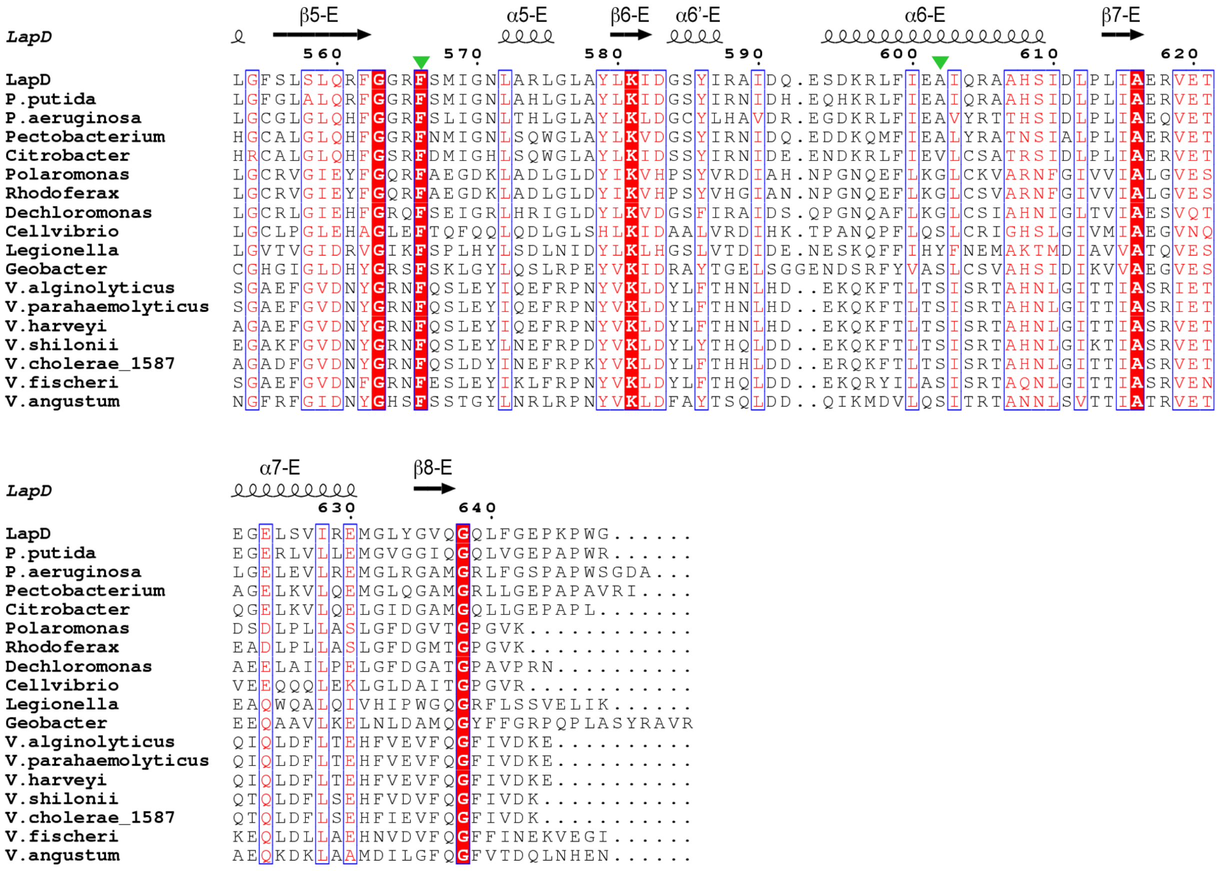
LapD
β5-E
α5-E
β6-E
α6'-E
α6-E
β7-E
560
570
580
590
600
610
620
LapD LGFSLSLQRFGGRFSMIGNLARLGLAYLKIDGSYIRAIDQ.ESDKRLFIEAIQRAAHSIDLPLIAERVET
P.putida LGFGLALQRFGGRFSMIGNLAHLGLAYLKIDGSYIRNIDH.EQHKRLFIEAIQRAAHSIDLPLIAERVET
P.aeruginosa LGCGLGLQHFGGRFSLIGNLTHLGLAYLKLDGCYLHAVDR.EGDKRLFIEAVYRTTHSIDLPLIAEQVET
Pectobacterium HGCALGLQHFGGRFNMIGNLSQWGLAYLKVDGSYIRNIDQ.EDDKQMFIEALYRATNSIALPLIAERVET
Citrobacter HRCALGLQHFGSRFDMIGHLSQWGLAYLKIDSSYIRNIDE.ENDKRLFIEVLCSATRSIDLPLIAERVET
Polaromonas LGCRVGIEYFGQRFAEGDKLADLGLDYIKVHPSYVRDIAH.NPGNQEFLKGLCKVARNFGIVVIALGVES
Rhodoferax LGCRVGIEYFGQRFAEGDKLADLGLDYIKVHPSYVHGIAN.NPGNQEFLKGLCSVARNFGIVVIALGVES
Dechloromonas LGCRLGIEHFGRQFSEIGRLHRIGLDYLKVDGSFIRAIDS.QPGNQAFLKGLCSIAHNIGLTVIAESVQT
Cellvibrio LGCLPGLEHAGLEFTQFQQLQDLGLSHLKIDAALVRDIHK.TPANQPFLQSLCRIGHSLGIVMIAEGVNQ
Legionella LGVTVGIDRVGIKFSPLHYLSDLNIDYLKLHGSLVTDIDE.NESKQFFIHYFNEMAKTMDIAVVATQVES
Geobacter CGHGIGLDHYGRSFSKLGYLQSLRPEYVKIDRAYTGELSGGENDSRFYVASLCSVAHSIDIKVVAEGVES
V.alginolyticus SGAEFGVDNYGRNFQSLEYIQEFRPNYVKLDYLFTHNLHD..EKQKFTLTSISRTAHNLGITTIASRIET
V.parahaemolyticus SGAEFGVDNYGRNFQSLEYIQEFRPNYVKLDYLFTHNLHD..EKQKFTLTSISRTAHNLGITTIASRIET
V.harveyi AGAEFGVDNYGRNFQSLEYIQEFRPNYVKLDYLFTHNLHD..EKQKFTLTSISRTAHNLGITTIASRVET
V.shilonii EGAKFGVDNYGRNFQSLEYLNEFRPDYVKLDYLYTHQLDD..EKQKFTLTSISRTAHNLGIKTIASRVET
V.cholerae_1587 AGADFGVDNYGRNFQSLDYINEFRPKYVKLDYLFTHHLDD..ERQKFTLTSISRTAHNLGITTIASRVET
V.fischeri SGAEFGVDNFGRNFESLEYIKLFRPNYVKLDYLFTHQLDD..EKQRYILASISRTAQNLGITTIASRVEN
V.angustum NGFRFGIDNYGHSFSSTGYLNRLRPNYVKLDFAYTSQLDD..QIKMDVLQSITRTANNLSVTTIATRVET
LapD
α7-E
β8-E
630
640
LapD EGELSVIREMGLYGVQGQLFGEPKPWG......
P.putida EGERLVLLEMGVGGIQGQLVGEPAPWR......
P.aeruginosa LGELEVLREMGLRGAMGRLFGSPAPWSGDA...
Pectobacterium AGELKVLQEMGLQGAMGRLLGEPAPAVRI....
Citrobacter QGELKVLQELGIDGAMGQLLGEPAPL.......
Polaromonas DSDLPLLASLGFDGVTGPGVK..........
Rhodoferax EADLPLLASLGFDGMTGPGVK..........
Dechloromonas AEELAILPELGFDGATGPAVPRN.........
Cellvibrio VEEQQQLEKLGLDAITGPGVR..........
Legionella EAQWQALQIVHIPWGQGRFLSSVELIK......
Geobacter EEQAAVLKELNLDAMQGYFFGRPQPLASYRAVR
V.alginolyticus QIQLDFLTEHFVEVFQGFIVDKE.........
V.parahaemolyticus QIQLDFLTEHFVEVFQGFIVDKE.........
V.harveyi QIQLDFLTEHFVEVFQGFIVDKE.........
V.shilonii QTQLDFLSEHFVDVFQGFIVDK..........
V.cholerae_1587 QTQLDFLSEHFIEVFQGFIVDK..........
V.fischeri KEQLDLLAEHNVDVFQGFFINEKVEGI......
V.angustum AEQKDKLAAMDILGFQGFVTDQLNHEN......
