# Supplementary figures and images for: Structural Basis for c-di-GMP-Mediated Inside-Out Signaling Controlling Periplasmic Proteolysis
Source: PLoS Biol. 2011 Feb 1;9(2):e1000588. doi: 10.1371/journal.pbio.1000588 (PMC3032553; doi:10.1371/journal.pbio.1000588)

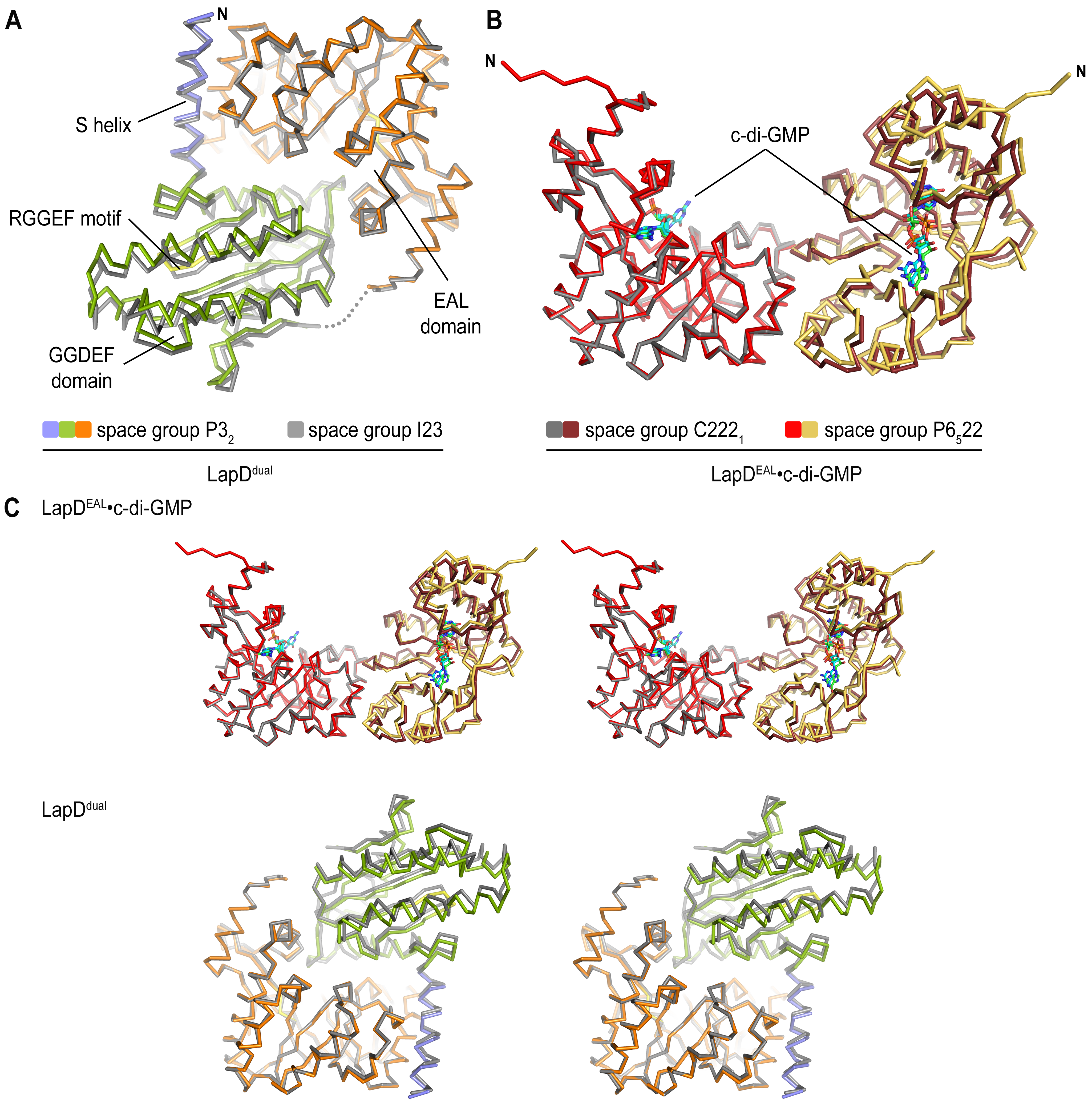

Supplement: Figure S2 — Crystal forms of LapDdual and LapDEAL•c-di-GMP. (A) LapDdual. Two independent crystal forms were obtained for LapDdual. The resulting structures were superimposed on the EAL domain and shown as protein backbone traces. (B) c-di-GMP-bound LapDEAL. Two independent crystal forms were obtained for LapDEAL. Both crystal lattices show the same dimeric assembly of EAL domains. Dimers were superimposed on one EAL domain and shown as protein backbone traces. (C) Stereo views. Stereo views of the structural comparisons shown in (A) and (B) are shown. In this view, the EAL domains of LapDdual and LapDEAL•c-di-GMP are shown in a similar orientation. (7.01 MB TIF) [file pbio.1000588.s002.tif]

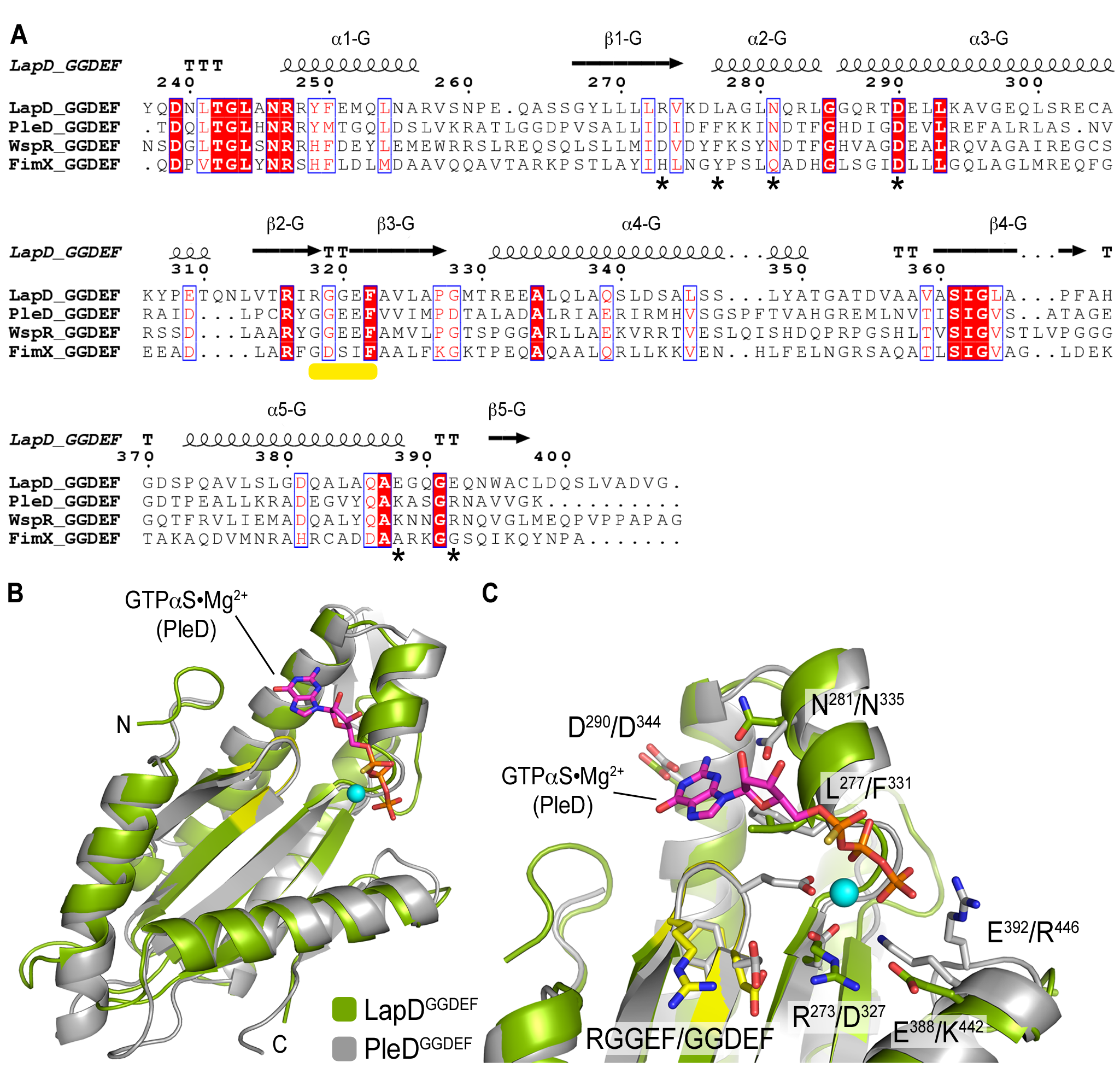

Supplement: Figure S3 — Comparison of the GGDEF domains from LapD and PleD. (A) Sequence alignment. Sequences of GGDEF domains with known structure were used to generate the alignment [17],[18],[36]. Conserved residues involved in nucleotide binding and hydrolysis are marked with asterisks [17],[38]. The GGDEF motif is highlighted with a yellow bar. (B) Overview. Structures of GGDEF domains of LapD and PleD (PDB ID 2v0n) are shown as a ribbon presentation [38]. A GTP analog bound to the active site of PleD is shown as a stick presentation. (C) GTP binding site. A close-up view of the active site is shown. Residues that in PleD are involved in nucleotide and divalent cation coordination are shown as a stick presentation. Left labels correspond to the LapD sequence; right labels correspond to the PleD sequence. (1.79 MB TIF) [file pbio.1000588.s003.tif]

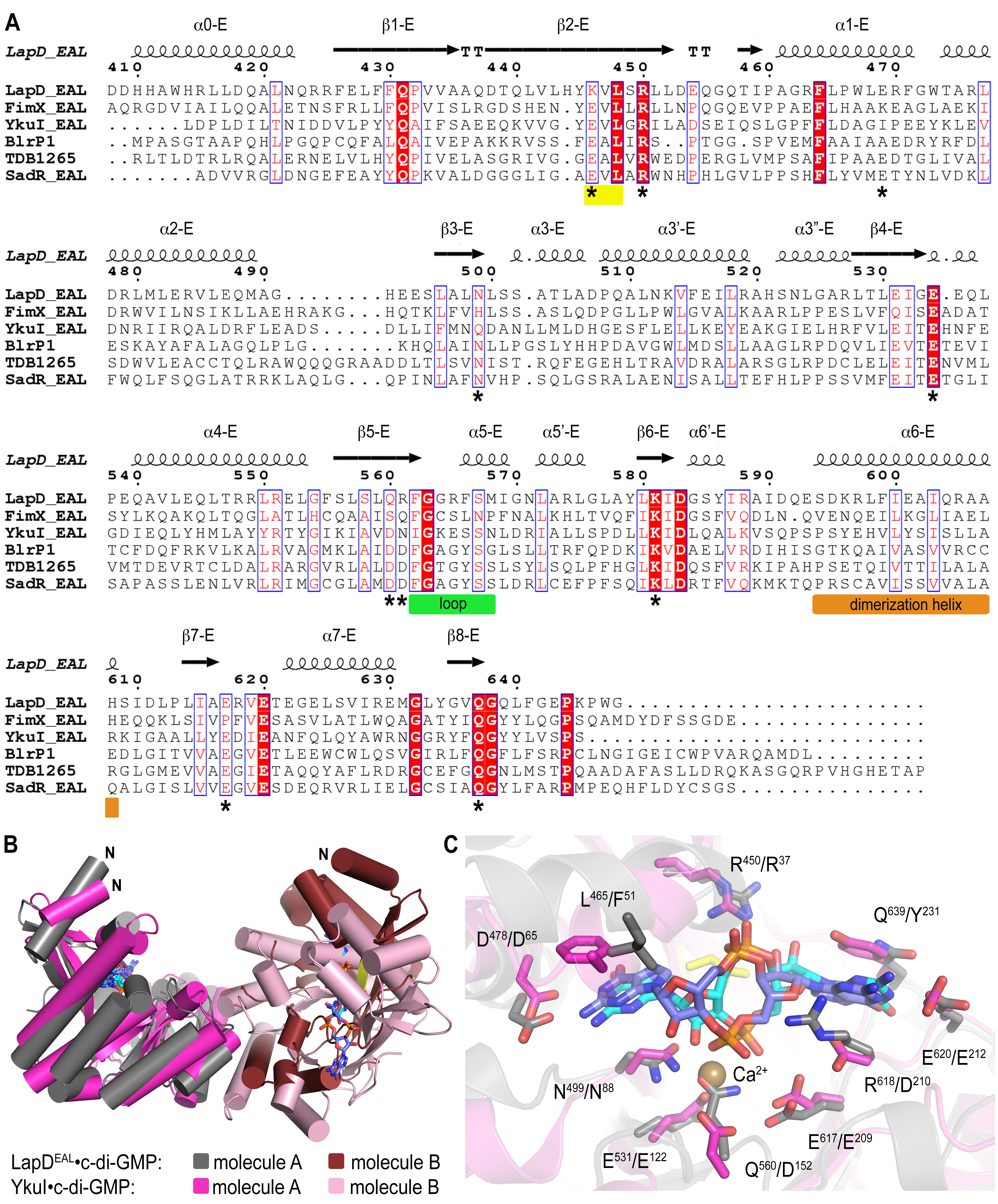

Supplement: Figure S4 — Comparison of c-di-GMP-bound LapDEAL and YkuI dimers. (A) Sequence alignment. Sequences of EAL domains with known structure were used to generate the alignment [34]–[37],[39]. Conserved residues in active phosphodiesterases are marked with asterisks [37]. The EAL motif is highlighted with a yellow bar. The loop and helix involved in dimerization are marked with a green and orange bar, respectively. (B) Overview. Structures of EAL domain dimers of LapD and YkuI bound to c-di-GMP (PDB ID 2w27) are shown as a ribbon presentation [35]. c-di-GMP is shown as a stick presentation. Structures were superimposed on one of the EAL domains of the dimeric assemblies. (C) c-di-GMP-binding site. A close-up view of the nucleotide-binding pocket is shown. Residues involved in c-di-GMP (and, in the case of YkuI, divalent cation) coordination are shown as a stick presentation. Left labels correspond to the LapD sequence; right labels correspond to the YkuI sequence. (7.34 MB TIF) [file pbio.1000588.s004.tif]

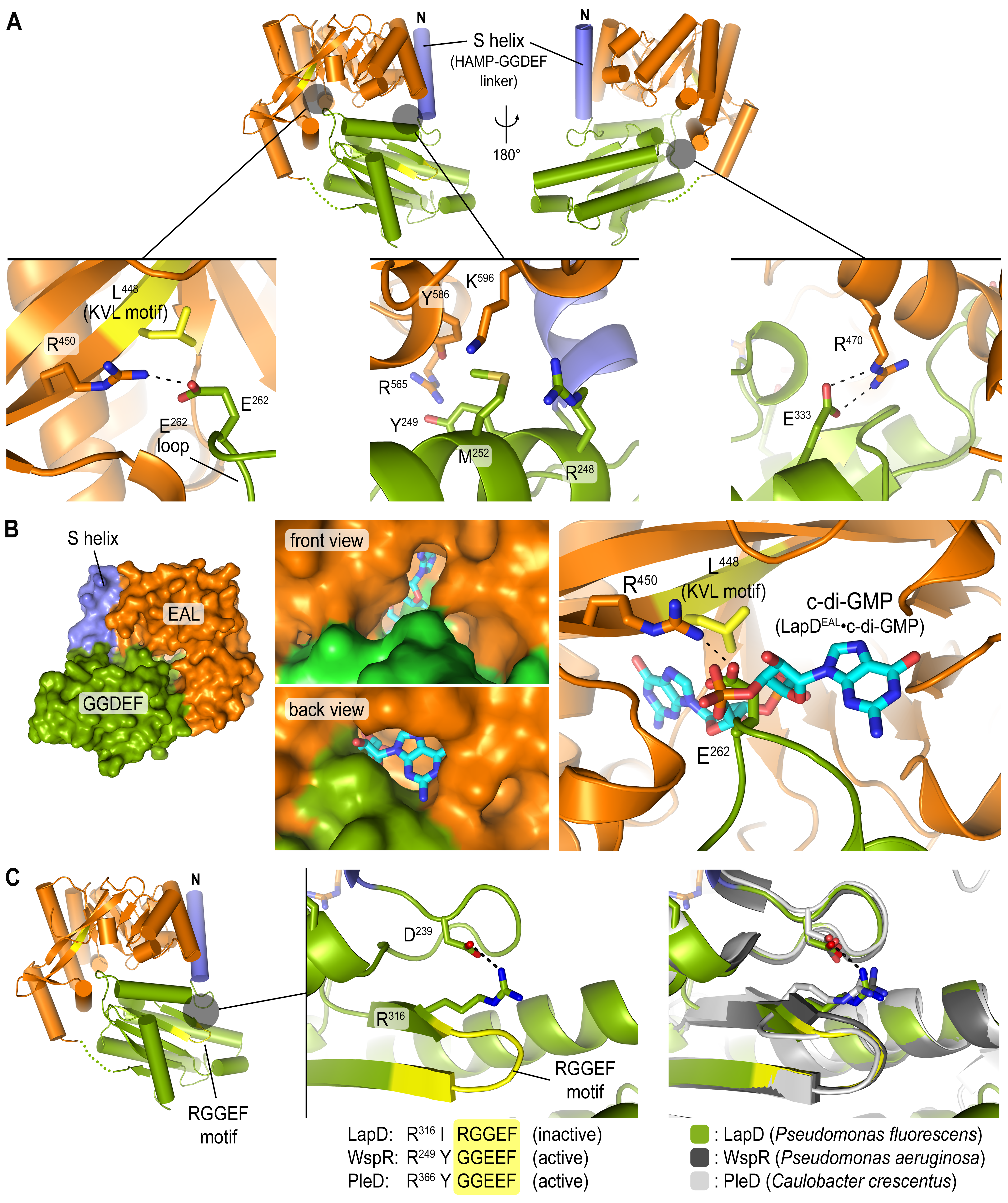

Supplement: Figure S5 — GGDEF–EAL domain interactions and S helix–GGDEF domain linker conformation observed in apo-LapDdual. (A) GGDEF–EAL domain interaction. Close-up views are shown for regions of direct contact between the GGDEF and EAL domains in the autoinhibited structure of LapDdual. The GGDEF and EAL domains are colored in green and orange, respectively. The S helix is colored in blue. (B) Nucleotide-binding pocket in apo-LapDdual. A close-up view of the c-di-GMP-binding pocket of LapD is shown (right panel). c-di-GMP is shown as a stick presentation after superimposing the crystal structure of LapDEAL•c-di-GMP onto the EAL domain of apo-LapDdual. The interacting residue pair R450/E262 in LapD is incompatible with c-di-GMP binding. The left panels show surface presentations of apo-LapDdual. The middle panel shows accessibility of the c-di-GMP-binding site, with c-di-GMP taken from LapDEAL•c-di-GMP after superimposition. (C) S helix–GGDEF connector. The S helix and the GGDEF domain are connected via a short loop that forms a tight turn. The loop conformation is conserved in other GGDEF domain–containing proteins, and is stabilized by the interaction between two residues D239 and R316, which are strictly conserved in many GGDEF domain–containing proteins [17],[18],[38],[41]. The arginine residue is directly preceding the GGDEF domain signature motif (GGDEF or GGEEF in active cyclases; RGGEF in LapD); the aspartate residue is located at the N-terminus of the loop. Its strict sequence and conformational conservation suggest a functional importance of the connector loop, likely restricting the conformational freedom between adjacent domains. (9.22 MB TIF) [file pbio.1000588.s005.tif]

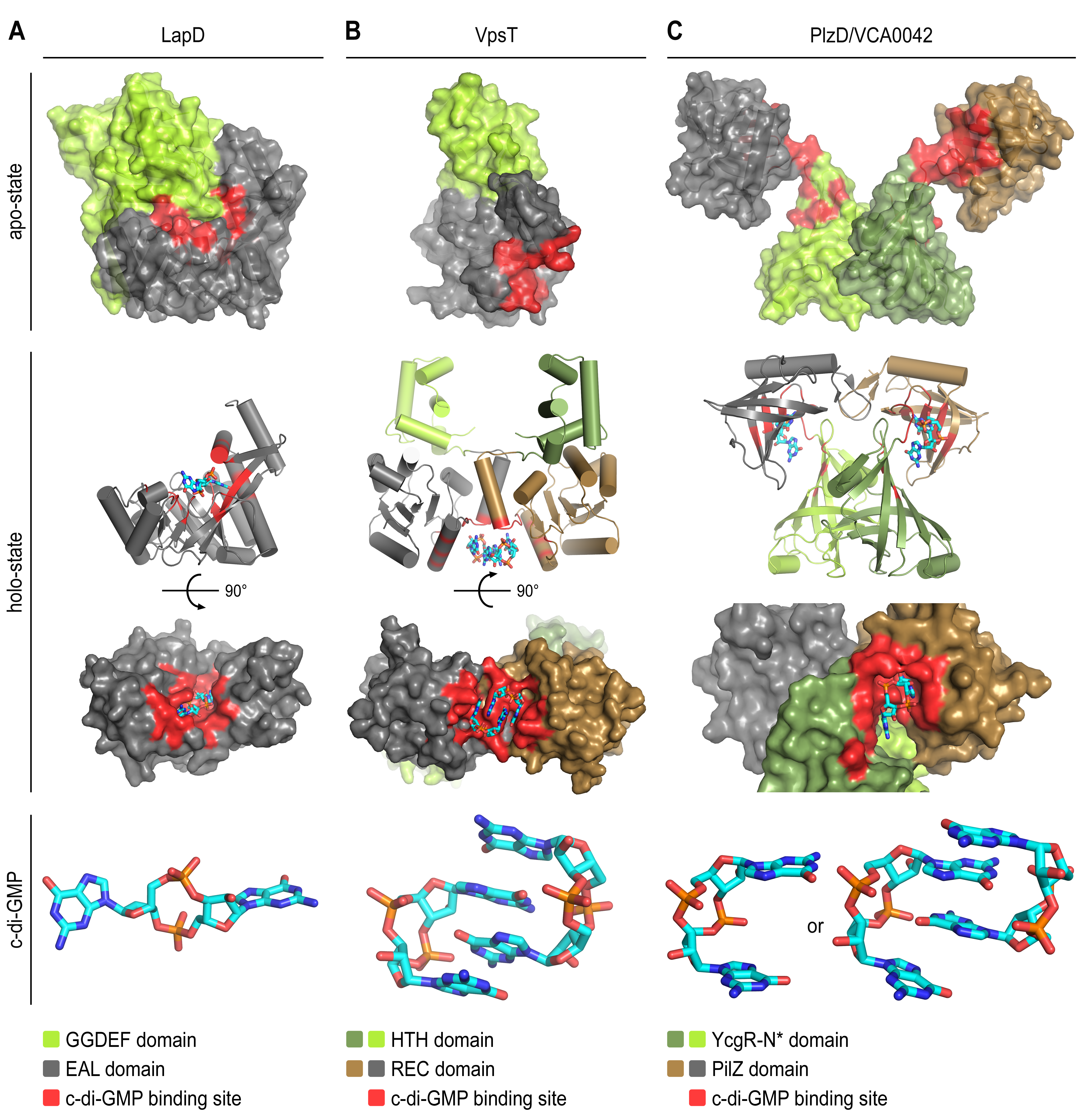

Supplement: Figure S6 — Structural comparion of LapD with other c-di-GMP receptors. (A) LapD. The monomoric apo-LapDdual structure is shown as a surface presentation (top). The middle panel shows the c-di-GMP-bound EAL domain of LapD in two orthogonal views. The dinucleotide-binding site is colored in red. The conformation of c-di-GMP is similar to that observed in other EAL domains such as FimX, YkuI, and BlrP1 (bottom panel) (see also Figure S4) [17],[18],[34]–[38]. (B) VpsT (PDB IDs 3kln and 3klo). The transcription factor VpsT from V. cholerae exists in a monomer–dimer equilibrium. An apo-VpsT monomer is shown as a surface presentation (top panel). The dimeric species is stabilized by c-di-GMP binding to the base of the regulatory receiver domain (middle panel) [14]. Two molecules of c-di-GMP form an intercalated dimer, similar to the binding mode observed for the inhibitory site binding in active diguanylate cyclases [17],[38]. The dinucleotide binding site is shown in red. (C) PilZ domains (PDB IDs 1yln, 2rde, 3yg, and 3kyf). The PliZ domain–containing protein PlzD/VCA0042 forms homodimers via its YcgR-N* domain. The PilZ domains form separate lobes of the protein. PilZ domain–containing proteins have been shown to bind either one or two mutually intercalated molecules of c-di-GMP [53],[54]. The dinucleotide-binding site is shown in red. (7.57 MB TIF) [file pbio.1000588.s006.tif]

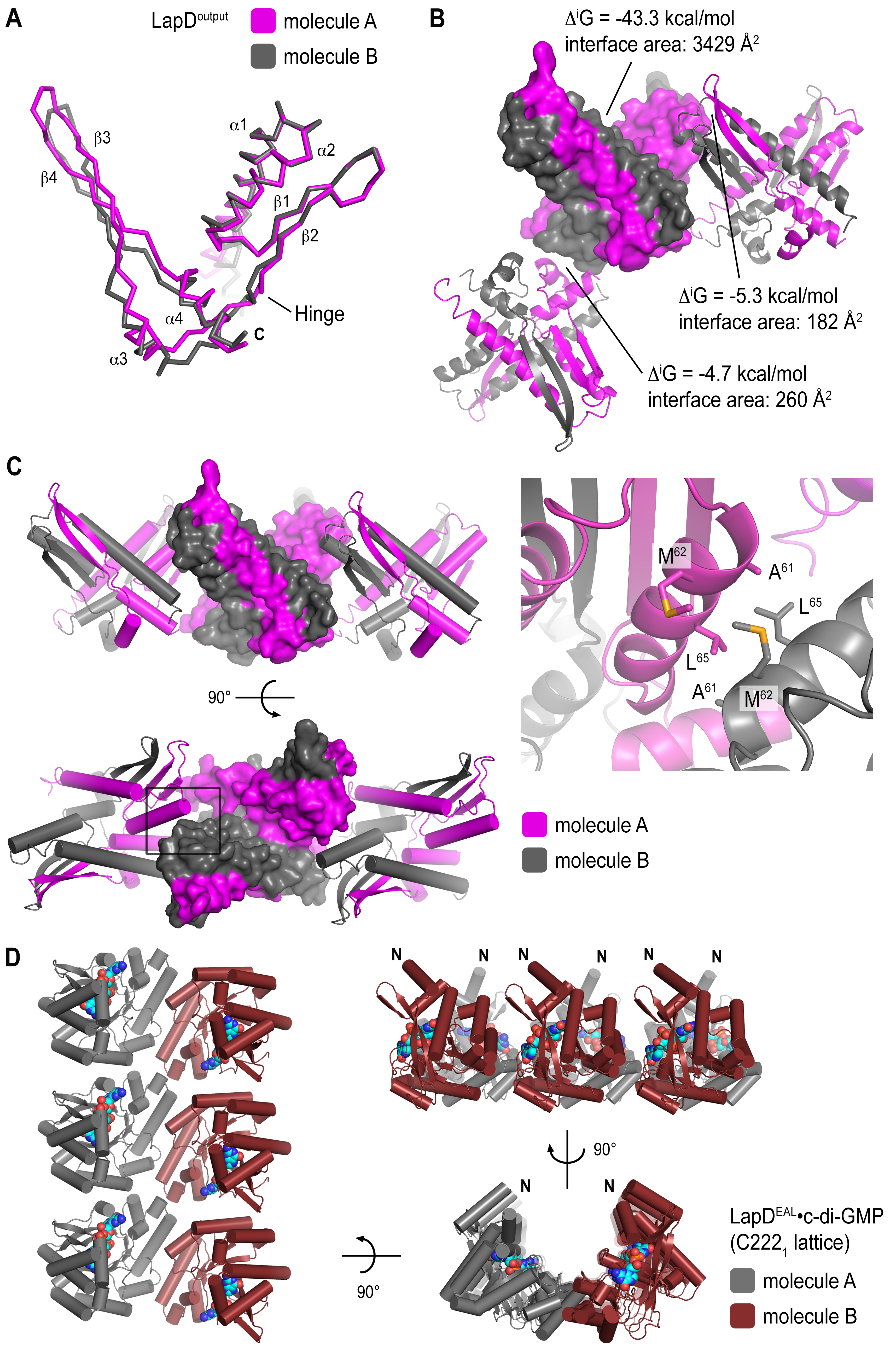

Supplement: Figure S7 — Structural analysis of LapDoutput and potential mechanisms for higher-order oligomerization of LapD. (A) Comparison between LapDoutput protomers. The periplasmic output domain of LapD crystallized with two molecules in the asymmetric unit. The protomers were superimposed on the first two helices of the fold, revealing a minor, rigid-body rotation of one half of the molecule relative to the other half between the two protomers. The rotation occurs at the connecting loop between β2 and α3 that forms the crossing-over point in the domain-swapped dimer. (B) LapDoutput crystal packing. Domain-swapped dimers of the output domain interact predominantly via two interfaces in the crystal lattices. One involves bottom-to-bottom interaction between LapDoutput dimers via a conserved, hydrophobic patch coinciding with the putative membrane-interaction surface. The other interface involves hydrophobic interactions between the arms of the V-shaped output domain dimers. (C) Potential higher-order oligomerization based on the structure of LapDoutput. Crystal lattice contacts reveal a potential mode for higher-order assemblies of LapD. The close-up view (right panel) shows the hydrophobic contacts between output domain dimers. (D) Potential higher-order oligomerization based on the structure of LapDEAL•c-di-GMP. In the C2221 crystal lattice, EAL domains form higher-order lattices that may highlight a mode for receptor oligomerization in the membrane. (7.15 MB TIF) [file pbio.1000588.s007.tif]

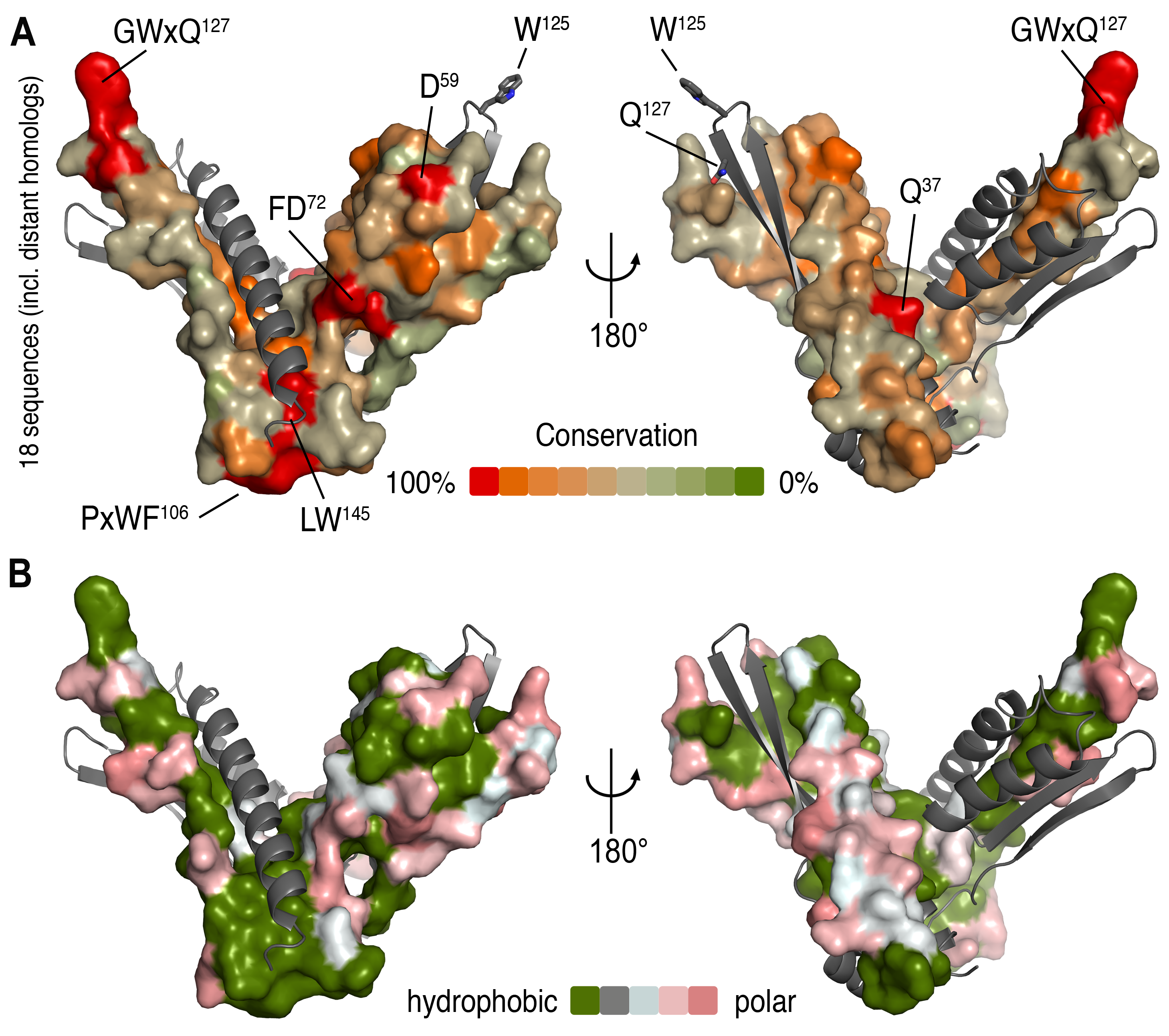

Supplement: Figure S8 — Surface conservation and hydrophobicity of LapDoutput. (A) Surface conservation. Based on an alignment of 18 sequences of LapD homologs (Figure S1), the sequence conservation was mapped onto the solvent-accessible surface of the output domain. One protomer is shown as a surface presentation, the other is shown as a ribbon presentation. Conserved motifs and individual residues are highlighted. Two views, separated by a 180° rotation, are shown. (B) Hydrophobicity mapped onto the molecular surface of LapDoutput. The surface is colored according to the hydrophobicity of accessible residues. Hydrophobic residues are shown in green; polar and charged residues are in gray and pink, respectively. (3.66 MB TIF) [file pbio.1000588.s008.tif]
