# Supplementary material for: Structural Basis for c-di-GMP-Mediated Inside-Out Signaling Controlling Periplasmic Proteolysis
Source: PLoS Biol. 2011 Feb 1;9(2):e1000588. doi: 10.1371/journal.pbio.1000588 (PMC3032553; doi:10.1371/journal.pbio.1000588)
Supplement: Table S1 — X-ray data collection and refinement statistics. (0.08 MB DOC) [file pbio.1000588.s009.doc]

**Table S1: X-ray data collection and refinement statistics.**

|  | LapD^EAL^•c-di-GMP (1) | LapD^EAL^•c-di-GMP (2) | LapD^output^ (Se-Met) | LapD^dual^ (1) | LapD^dual^ (2) | LapD^dual^ (2) (Se-Met) |
| --- | --- | --- | --- | --- | --- | --- |
| **Data Collection** | | | | | | |
| X-ray source | CHESS, A1 | CHESS, A1 | CHESS, A1 | CHESS, F2 | CHESS, A1 | CHESS, A1 |
| Wavelength (Å) | 0.9771 | 0.9771 | 0.9771 | 0.9793 | 0.9771 | 0.9771 |
| Space group | C222_1_ | P6_5_22 | P2_1_2_1_2_1_ | I23 | P3_2_ | P3_2_ |
| Unit cell |  |  |  |  |  |  |
| a, b, c (Å) | 41.4, 204.8, 142.4 | 141.5, 141.5, 111.2 | 41.5, 71.4, 110.4 | 154.8, 154.8, 154.8 | 52.1, 52.1, 146.3 | 52.4, 52.4, 146.5 |
| α, β, γ (°) | 90, 90, 90 | 90, 90, 120 | 90, 90, 90 | 90, 90, 90 | 90, 90, 120 | 90, 90, 120 |
| Resolution (Å)^a^ | 50-2.5 (2.59-2.5) | 50-2.5 (2.59-2.5) | 50-1.8 (1.83-1.77) | 50-3.1 (3.29-3.1) | 30-2.0 (2.07-2.0) | 50-2.5 (2.59-2.5) |
| No. of reflections |  |  |  |  |  |  |
| Total | 172,322 (12,035) | 568,367 (34,934) | 433,383 (26,900) | 123,220 (18,906) | 188,981 (17,162) | 105,992 (8,859) |
| Unique | 20,943 (1,973) | 23,191 (2,211) | 32,517 (3,092) | 11,246 (1,775) | 29,997 (2,959) | 31,174 (3,055) |
| Completeness (%) | 99.1 (94.4) | 99.7 (97.4) | 99.5 (96.7) | 98.9 (98.7) | 99.7 (98.8) | 99.8 (98.4) |
| Redundancy | 8.2 (6.1) | 24.5 (15.8) | 13.3 (8.7) | 10.9 (10.7) | 6.3 (5.8) | 3.4 (2.9) |
| I/σ(I) | 19.8 (3.5) | 34.4 (3.7) | 30.2 (4.1) | 29.4 (3.4) | 42.6 (3.3) | 24.0 (3.2) |
| R_meas_ (%) | 8.7 (33.9) | 7.5 (43.6) | 6.5 (37.3) | 7.8 (78.5) | 4.4 (47.7) | 4.7 (28.2) |
| **Refinement** | | | | | | |
| R_work_ / R_free_ (%) | 18.1 / 24.5 | 19.6 / 22.5 | 19.4 / 22.5 | 23.4 / 28.4 | 18.1 / 22.2 |  |
| rms deviations |  |  |  |  |  |  |
| Bond length (Å) | 0.009 | 0.008 | 0.006 | 0.011 | 0.011 |  |
| Bond angles (°) | 1.271 | 1.239 | 0.994 | 1.405 | 1.233 |  |
| No. of atoms |  |  |  |  |  |  |
| Protein | 3866 | 2001 | 1979 | 3315 | 3315 |  |
| c-di-GMP | 92 | 46 | 0 | 0 | 0 |  |
| Water | 115 | 125 | 304 | 0 | 180 |  |
| Ave. B-factors (Å^2^) |  |  |  |  |  |  |
| Protein | 37.2 | 54.2 | 32.6 | 83.8 | 54.0 |  |
| cyclic di-GMP | 28.4 | 46.9 | -- | -- | -- |  |
| Water | 34.3 | 50.6 | 43.3 | -- | 51.1 |  |
| Ramachandram (%) |  |  |  |  |  |  |
| Favored | 93.2 | 93.2 | 94.3 | 88.5 | 93.6 |  |
| Allowed | 6.8 | 6.8 | 5.7 | 11.2 | 5.8 |  |
| Generously allowed | 0.0 | 0.0 | 0.0 | 0.0 | 0.3 |  |
| Disallowed | 0.0 | 0.0 | 0.0 | 0.3 | 0.3 |  |

(a) Values in brackets are for the highest resolution bin.
