# Supplementary material for: Structural Basis for c-di-GMP-Mediated Inside-Out Signaling Controlling Periplasmic Proteolysis
Source: PLoS Biol. 2011 Feb 1;9(2):e1000588. doi: 10.1371/journal.pbio.1000588 (PMC3032553; doi:10.1371/journal.pbio.1000588)
Supplement: Table S2 — Strains and plasmids. (0.05 MB DOC) [file pbio.1000588.s010.doc]

**Table S2: Strains and plasmids.**

| **Strain or plasmid** | **Genotype or Description** | **Reference** |
| --- | --- | --- |
| ***Escherichia coli*** |  |  |
| S17-1(λpir) | *thi pro hsdR- hsdM+* Δ*recA* RP4-2::TcMu-Km::Tn7 | (Simon et al*.*, 1983) |
| ***Saccharomyces cerevisiae*** |  |  |
| InvSc1 | uracil auxotroph | Invitrogen |
| ***Pseudomonas fluorescens*** |  |  |
| Δ*lapD* | unmarked deletion of the *lapD* gene | (Newell et al., 2009) |
|  |  |  |
| **Plasmids** |  |  |
| pMQ72 | *2μ URA3; ori & rep* pR01600; *colE1*, *aac1* *P_BAD_ araC* | (Shanks et al., 2006) |
| pLapD | LapD with C-terminal 6 His tag expressed from *P_BAD_* | (Newell et al., 2009) |
| pLapD ΔH1 | pLapD with 7 amino acid deletion in first helix of HAMP domain | (Newell et al., 2009) |
| pLapD W125E | pLapD point mutation in the indicated codon or codons | This study |
| pLapD W125E,S229D | pLapD point mutation in the indicated codon or codons | This study |
| pLapD F222A | pLapD point mutation in the indicated codon or codons | This study |
| pLapD F222E | pLapD point mutation in the indicated codon or codons | This study |
| pLapD S229D | pLapD point mutation in the indicated codon or codons | This study |
| pLapD E230A | pLapD point mutation in the indicated codon or codons | This study |
| pLapD L232E | pLapD point mutation in the indicated codon or codons | This study |
| pLapD M252E | pLapD point mutation in the indicated codon or codons | This study |
| pLapD E262A | pLapD point mutation in the indicated codon or codons | This study |
| pLapD E333A | pLapD point mutation in the indicated codon or codons | This study |
| pLapD A602E | pLapD point mutation in the indicated codon or codons | This study |
| pLapD F222E,A602E | pLapD point mutation in the indicated codon or codons | This study |
| pLapD S229D,A602E | pLapD point mutation in the indicated codon or codons | This study |
