# Supplementary material for: Structural Basis for c-di-GMP-Mediated Inside-Out Signaling Controlling Periplasmic Proteolysis
Source: PLoS Biol. 2011 Feb 1;9(2):e1000588. doi: 10.1371/journal.pbio.1000588 (PMC3032553; doi:10.1371/journal.pbio.1000588)
Supplement: Text S1 — Supporting information. The file includes supplemental Materials and Methods and associated references. (0.07 MB DOC) [file pbio.1000588.s011.doc]

**Supporting Information**

**Supplemental Materials and Methods**

**Protein expression and purification**

For *in vivo* experiments, LapD protein variants were expressed from an arabinose-inducible vector (pMQ72) in a Δ*lapD* strain as previously described [1]. Proteins used in crystallization and *in vitro* studies were expressed and purified as follows.

Construct boundaries were chosen based on secondary structure predictions and sequence alignments. The coding regions corresponding to the GGDEF-EAL dual domain module (LapD^dual^; residues 220-648) and to the isolated EAL domain (LapD^EAL^; residues 399-648) of *P. fluorescens* Pf0-1 LapD were amplified by standard PCR and cloned into a modified pProExHtb expression vector (Invitrogen), where the TEV protease cleavage site was engineered into a PreScission Protease site for removal of the N-terminally fused hexahistidine (His_6_) moiety. The coding region corresponding to the periplasmic output domain of LapD (LapD^output^; residues 22-151) was PCR-amplified and cloned into a modified pET28a expression plasmid (Novagen) yielding an N-terminally His_6_-tagged SUMO fusion protein. The His_6_-tagged SUMO moiety was cleavable using the yeast protease Ulp-1. Finally, LapG protein was expressed as a C-terminally His_6_-tagged version after cloning and expression from a pET21a expression vector (Novagen).

Native and selenomethionine-derivatized proteins were overexpressed in *E. coli*, as previously described [2]. Briefly, native proteins were expressed in T7 Express cells (NEB), grown at 37ºC in Terrific Broth (TB) media supplemented with 50 μg/ml kanamycin for the pET28a vector, or 100 μg/ml ampicillin for the pProEx and pET21 vectors. At an optical density corresponding to 0.8-1.2 absorbance at 600 nm (OD_600_), the temperature was reduced to 18ºC and protein expression was induced with 1 mM IPTG. Selenomethionine-derivatized proteins were expressed in T7 Crystal Express cells (NEB), grown in M9 minimal media supplemented with the appropriate antibiotic, vitamins (1 μg/ml thiamin and 1 μg/ml biotin), carbon source (0.4% glucose), trace elements, and amino acids (40 μg/ml of each of the 20 amino acids with selenomethionine substituting for methionine). Protein expression in minimal medium was induced at cell densities corresponding to OD_600_ of 0.4-0.5. After 16 hours of expression at 18 degrees, cells were harvested by centrifugation, resuspended in NiNTA buffer A (25 mM Tris-HCl, pH 8.4, 500 mM NaCl, and 20 mM Imidazole), and flash-frozen in liquid nitrogen.

After thawing and cell lysis by sonication, cellular debris were removed by centrifugation and clear lysates were incubated with NiNTA resin (Qiagen) equilibrated in NiNTA buffer A. The resin was washed excessively with buffer A and proteins were eluted in a single step of NiNTA buffer A supplemented with 500 mM Imidazole. Proteins were buffer exchanged into desalting buffer (25 mM Tris-HCl, pH 8.4, 300 mM NaCl, and 5 mM β-mercaptoethanol) and, where applicable, affinity tags were removed by incubation with the yeast protease Ulp-1 or PreScission Protease at 4ºC overnight. Cleaved proteins were collected in the flow-through during a second step of NiNTA affinity chromatography (HisTrap; GE Healthcare). As a final step of protein purification, proteins were concentrated and subjected to size-exclusion chromatography on a Superdex200 column (GE Healthcare) equilibrated with gel filtration buffer (25 mM Tris-HCl, pH 8.4, and 250-300 mM NaCl). Proteins were concentrated on a Centricon ultracentrifugation device with an appropriate molecular weight cut-off (Millipore) to final concentrations in the low milimolar range. Protein aliquots were flash frozen in liquid nitrogen and stored at -80ºC.

**Crystallization, X-ray data collection, and structure solution**

All crystals were obtained by hanging drop vapor diffusion after mixing equal volumes of protein (10-30 mg/ml) and reservoir solution. LapD^dual^ was crystallized without cleavage of the N-terminally fused hexahistidine tag. Native and selenomethionine-derivatized proteins yielded single crystals grown at 20ºC with reservoir solution containing 14% PEG 4000 and 0.1 M MES, pH 6.0 (space group P3_2_). In addition, LapD^dual^ yielded crystals with I23 space group symmetry after mixing with reservoir solution of 0.2 M Ammonium acetate, 0.1 M Sodium citrate, pH 5.6, and 15% PEG 4000. For cryoprotection in either case, crystals were soaked in reservoir solution supplemented with 30% Ethylene glycol prior to freezing.

For crystallization of the isolated, untagged EAL domain in the presence of c-di-GMP, the protein solution was supplemented with 1 mM purified nucleotide prior to setting up the crystallization trials. Diffraction-quality crystals with P6_5_22 space group symmetry grew after incubation for 7-10 days at 4ºC with well solution containing 0.1 M Bis-Tris, pH 6.5, and 1.5 M Ammonium sulfate. Cyclic di-GMP bound EAL domain crystals with C222_1_ symmetry grew at 20ºC in a crystallization condition containing 0.1 M Bis-Tris, pH 5.5, 0.2 M Ammonium sulfate, and 24% PEG 3350. Prior to freezing, the crystals were soaked in their respective reservoir solutions supplemented with 25% of the cryoprotectant xylitol.

LapD^output^ crystals used for data collection were grown at 4ºC after mixing with a reservoir solution containing 22% PEG monomethyl ether 2,000 and 0.15 M Potassium bromide. For crystal freezing, the mother liquor was supplemented with 20% xylitol to ensure cryoprotection.

Cryo-preserved crystals for all protein constructs were flash-frozen and stored in liquid nitrogen. Data were collected on frozen crystals at 100K using synchrotron radiation at the Cornell High Energy Synchrotron Source (CHESS).

Data reduction was carried out with the software package HKL2000 [3] and XDS [4]. Experimental phases for LapD^dual^ and LapD^output^ crystals were obtained from Single Anomalous Diffraction (SAD) experiments on crystals grown from selenomethionine-derivatized proteins. Heavy atom positions and solvent flattening was carried out by using the software package PHENIX [5]. For LapD^dual^, initial phases were extended by using the software DM [6], and the first model was built into the electron density map automatically by using the software Buccaneer [7]. The structure of nucleotide-bound LapD^EAL^ was determined by molecular replacement in PHENIX with the EAL domain of LapD^dual^ as the search model. Refinement in PHENIX and COOT yielded the final models [5,8]. Data collection and refinement statistics are summarized in Table S1. Illustrations were made in Pymol (DeLano Scientific).

**Enzymatic production of c-di-GMP**

Cyclic di-GMP used in crystallization trials and c-di-GMP binding assays was synthesized enzymatically using a constitutively active WspR mutant (PA3702 R^242^A) and GTP as a substrate [9]. Following purification by preparative reverse-phase HPLC and lyophilization, the nucleotide product was enzymatically tested as a substrate for phosphodiesterases (data not shown). Cyclic di-GMP purity and concentration were determined based on absorbance at 254 nm in comparison with commercially obtained standards (Biolog Life Science Institute).

**Construction of LapD variants**

For *in vitro* studies, *ladD* point mutants were generated using a QuikChange site directed mutagenesis kit (Stratagene) following the manufacturer’s instructions. For cell-based studies, *lapD* alleles were generated by recombination cloning [10]. Briefly, *lapD* was amplified in two pieces, the 5’ fragment ending just upstream of the codon to be changed and the 3’ fragment beginning just downstream. The new codon and 21 complimentary bases were included in each internal primer such that recombination in yeast with the parent pMQ72 vector would yield a contiguous *lapD* ORF with the desired codon change.

**Supplemental References**

1. Newell PD, Monds RD, O'Toole GA (2009) LapD is a bis-(3',5')-cyclic dimeric GMP-binding protein that regulates surface attachment by *Pseudomonas fluorescens* Pf0-1. Proc Natl Acad Sci U S A 106: 3461-3466.

2. Krasteva PV, Fong JC, Shikuma NJ, Beyhan S, Navarro MV, et al. (2010) *Vibrio cholerae* VpsT regulates matrix production and motility by directly sensing cyclic di-GMP. Science 327: 866-868.

3. Otwinowski Z, Minor W (1997) Processing of X-ray diffraction data collected in oscillation mode. Methods Enzymol 276: 307-326.

4. Kabsch W (2010) Xds. Acta Crystallogr D Biol Crystallogr 66: 125-132.

5. Adams P, Grosse-Kunstleve R, Hung L, Ioerger T, McCoy A, et al. (2002) PHENIX: building new software for automated crystallographic structure determination. Acta Crystallogr D Biol Crystallogr 58: 1948-1954.

6. Cowtan K (1994) 'dm': An automated procedure for phase improvement by density modification. Joint CCP4 and ESF-EACBM Newsletter on Protein Crystallography 31: 34-38.

7. Cowtan K (2006) The Buccaneer software for automated model building. 1. Tracing protein chains. Acta Crystallogr D Biol Crystallogr 62: 1002-1011.

8. Emsley P, Cowtan K (2004) Coot: model-building tools for molecular graphics. Acta Crystallographica Section D-Biological Crystallography 60: 2126-2132.

9. De N, Pirruccello M, Krasteva PV, Bae N, Raghavan RV, et al. (2008) Phosphorylation-independent regulation of the diguanylate cyclase WspR. PLoS Biol 6: e67.

10. Shanks RM, Caiazza NC, Hinsa SM, Toutain CM, O'Toole GA (2006) *Saccharomyces cerevisiae*-based molecular tool kit for manipulation of genes from gram-negative bacteria. Appl Environ Microbiol 72: 5027-5036.
